# Opening Diffusion Pathways through Site Disorder: The Interplay of Local Structure and Ion Dynamics in the Solid Electrolyte Li_6+*x*_P_1–*x*_Ge_*x*_S_5_I as Probed by Neutron Diffraction and NMR

**DOI:** 10.1021/jacs.1c11571

**Published:** 2022-01-20

**Authors:** Katharina Hogrefe, Nicolò Minafra, Isabel Hanghofer, Ananya Banik, Wolfgang G. Zeier, H. Martin R. Wilkening

**Affiliations:** † Institute of Chemistry and Technology of Materials, Graz University of Technology (NAWI Graz), Stremayrgasse 9, A-8010 Graz, Austria; ‡ Institute of Inorganic and Analytical Chemistry, University of Münster, Correnstrasse 30, D-48149 Münster, Germany; § Institut für Energie- und Klimaforschung (IEK), IEK-12: Helmholtz-Institut Münster, Forschungszentrum Jülich, Corrensstrasse 46, 48149 Münster, Germany

## Abstract

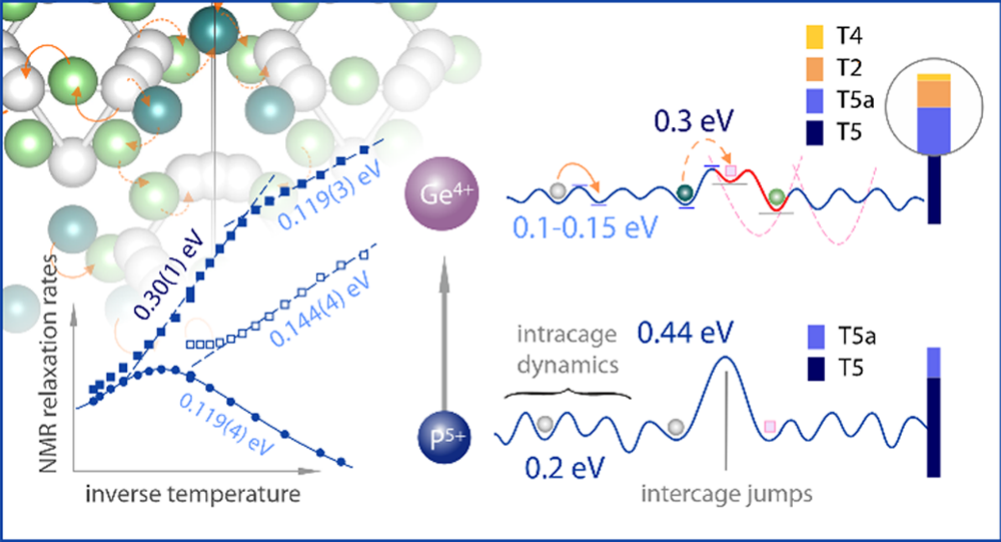

Solid electrolytes are at the heart of future energy storage systems. Li-bearing argyrodites are frontrunners in terms of Li^+^ ion conductivity. Although many studies have investigated the effect of elemental substitution on ionic conductivity, we still do not fully understand the various origins leading to improved ion dynamics. Here, Li_6+*x*_P_1–*x*_Ge_*x*_S_5_I served as an application-oriented model system to study the effect of cation substitution (P^5+^ vs Ge^4+^) on Li^+^ ion dynamics. While Li_6_PS_5_I is a rather poor ionic conductor (10^–6^ S cm^–1^, 298 K), the Ge-containing samples show specific conductivities on the order of 10^–2^ S cm^–1^ (330 K). Replacing P^5+^ with Ge^4+^ not only causes S^2–^/I^–^ anion site disorder but also reveals via neutron diffraction that the Li^+^ ions do occupy several originally empty sites between the Li rich cages in the argyrodite framework. Here, we used ^7^Li and ^31^P NMR to show that this Li^+^ site disorder has a tremendous effect on both local ion dynamics and long-range Li^+^ transport. For the Ge-rich samples, NMR revealed several new Li^+^ exchange processes, which are to be characterized by rather low activation barriers (0.1–0.3 eV). Consequently, in samples with high Ge-contents, the Li^+^ ions have access to an interconnected network of pathways allowing for rapid exchange processes between the Li cages. By (i) relating the changes of the crystal structure and (ii) measuring the dynamic features as a function of length scale, we were able to rationalize the microscopic origins of fast, long-range ion transport in this class of electrolytes.

## Introduction

The demand for electrochemical energy storage systems has reached an unprecedented level to power electrical vehicles or to store electricity for electricity grids. Li-based batteries represent one of the most advanced storage systems to meet these needs. To increase both performance and safety of existing systems, so-called all-solid-state batteries are currently developed. In the case of batteries taking advantage of a ceramic electrolyte, plenty of workgroups are feverishly searching for new materials with outstanding Li^+^ conduction properties, which are at the heart of such devices.^1−4^ Hence, rationalizing the underlying principles governing Li^+^ ion transport is key in designing powerful and long-lasting ceramic batteries. The right materials may also help stabilizing the various macroscopic interfaces in these multicomponent systems.

Within the class of inorganic electrolytes, Li-containing thiophosphates these days feature the highest ionic conductivities.^5,6^ In particular, the conduction properties of Li argyrodites with the general formula Li_6_PS_5_X (X = Cl, Br, and I) have been the subject of plenty of studies as their specific ionic conductivities reach values up to the range of several mS cm^–1^ at room temperature.^7−12^ In some of these cases, they even exceed the benchmark of 10 mS cm^–1^, thus outperforming some of the liquid electrolytes used in classical Li-ion batteries.

As suggested by several studies, cation and anion site disorder seem to be the cause of the extremely high ion conductivities observed in, for example, disordered Li_6_PS_5_Br. Anion site disorder means that different anions, also differing in ionic radii, occupy the same crystallographic site in a random order. In contrast, Li^+^ site disorder is present in samples where the Li^+^ ions do randomly occupy various crystallographically inequivalent sites leading to partially filled Li^+^ sublattices. For instance, in Li_6_PS_5_Br the extent of anion S^2–^/Br^–^ site disorder was successfully manipulated by “kinetic freezing”.^10,13^ Another approach, also leading to a higher degree of anion mixing, was pursued by Wang et al.^14^ The authors increased the bromine content and prepared Li_6–*x*_PS_5–*x*_Br_1+*x*_, a Li-deficient compound. In another strategy, the lithium content was increased by substituting Si for P yielding Li_6+*x*_P_1–*x*_Si_*x*_S_5_Br with increased ionic conductivity compared to the unsubstituted sample.^15^ Furthermore, a very recent study evaluated aliovalent cation substitution in Cl-rich argyrodites.^16^

Considering Li_6_PS_5_I, the replacement of pentavalent P^5+^ through tetravalent Ge^4+^ leads to Li_6+*x*_P_1–*x*_Ge_*x*_S_5_I with an enhanced Li content.^17^ Similarly, Zhou et al. studied cation and anion site disorder in Li_6+*x*_M_*x*_Sb_1–*x*_S_5_I compounds with M = Si, Ge, and Sn.^18^ The substitution effect in Li_6+*x*_P_1–*x*_Ge_*x*_S_5_I was already studied by impedance spectroscopy, X-ray diffraction, and nuclear magnetic resonance.^17^ At sufficiently high Ge content, the samples show increased anion site disorder and reveal higher ionic conductivities associated with lower activation barriers. The key origins leading to increased Li^+^ exchange processes and facile long-range ion transport are, however, not yet fully understood.

We can think of several adjusting screws^12,19−21^ to influence Li^+^ cation translational dynamics on both the short- and long-range length scales.^22−24^ Concepts include, for example, the discussion of structural parameters and polyhedra connections, polarization effects, and the change of Li–S bond strengths through both alio- and isovalent substitution. Anion site disorder might also influence the Li^+^ sublattice as originally empty (interstitial) Li^+^ sites become occupied in anion-mixed compounds. The various effects will affect the underlying potential energy landscape to variable extents. In the most general sense, the local landscape or energy potential of a given compound with fixed composition should be considered as a dynamic rather than a static one as, for instance, (i) the localized hopping of positive charge through correlated motions or forward–backward jumps^25,26^ and/or (ii) any rotational motions of the polyanions might render this landscape time-dependent. This view is in contrast to that of a rigid lattice framework with the Li^+^ cations being regarded as random walkers carrying out completely uncorrelated jump processes with negligible lattice relaxation.^26^ Recently, Weitzel and co-workers significantly advanced this field by analyzing site energy distributions and energy landscapes in a variety of crystalline and amorphous solids.^27−30^

In particular, for crystalline solids providing structures with mobile ions and stationary polyanions the correlation between translational and rotational motion and its implications on ionic transport is a vividly discussed topic. Mechanisms like the so-called “paddle-wheel” or “cogwheel” effect^31−34^ were used to illustratively describe the coupled motion of polyanion rotation with the translation of mobile species that finally leads to high ionic conductivity in so-called rotor phases. Usually, this effect is seen at rather high temperatures. Experimentally, it is often difficult to present a conclusive proof for one of these mechanisms. In a very recent example, Smith and Siegel^35^ investigated the correlation of Li^+^ translation and PS_4_^3–^ rotational dynamics in glassy and crystalline Li_3_PS_4_ by *ab initio* molecular dynamics (AIMD) simulations. Indeed, they found facilitated PS_4_^3–^ rotations for the glassy, that is, the amorphous, phase and linked this feature to the higher Li^+^ ion translational dynamics. In the crystalline form, they found anion reorientations to be negligible. Moreover, differences in Li^+^ dynamics were explained by distinct densities and long-range covalent networks in the crystalline and glassy structures.^35^ Zhang et al.^36^ used results from maximum entropy method analysis, AIMD and joint-time correlation analysis to describe rotational motion in Na_11_Sn_2_PnS_12_ (Pn = P and Sb). However, the presented “universal design principle for fast ion conductors” also includes the case for Na_11_Sn_2_SbS_12_, where simulations show no SbS_4_^3–^ rotational motions, but the Na^+^ ionic conductivity is lower only by a factor of 10 than in the rotor phases including phosphorus.^37^ Thus, the extent of rotational motions on translational cation dynamics needs further investigation. Notably, as also found for the Li_3_PS_4_ glass phase, the power spectrum of both anions and cations in Na_11_Sn_2_PnS_12_ (Pn = P and Sb) strongly overlap, which the authors interpret as the occurrence of both processes at the same time scale.^35,37^

In the case of Li_6_PS_5_X, we used ^7^Li and ^31^P NMR to directly sense the different motion-controlled fluctuations the nuclei are exposed to in compounds with X = Cl, Br, and I. For Li_6_PS_5_I, the ^31^P NMR spin–lattice relaxation rates (1/*T*_1_) at low temperature were interpreted as a result of fast PS_4_^3–^ rotational motions.^11^ However, the rotational jumps seem to be decoupled from any Li^+^ translational dynamics, as PS_4_^3–^ dynamics proceeds on a much shorter time scale as Li^+^ translation. Li_6_PS_5_I is known to be a rather poor ionic conductor (10^–6^ S cm^–1^ at room temperature).^7,38^ Interestingly, increasing site disorder and lattice contraction slow down these rotations.^11^ Most importantly, in Li_6_PS_5_Br, dynamic coupling of the rotational jumps and the Li^+^ cation translational movements were found to occur on the same time-scale, thus strongly pointing to an interplay of these two dynamic processes.^11^ In nanocrystalline Li_6_PS_5_I prepared by ball-milling, enhanced Li^+^ translational dynamics are mainly traced back to increased Li^+^ and I^–^/S^2–^ site disorder.^38,39^ Once again, structural disorder, possibly also including site disorder, slows down ^31^P NMR spin–lattice relaxation in nano-Li_6_PS_5_I.^38,40^

In the present study, we used a combination of ^7^Li and ^31^P NMR spin–lattice relaxation (SLR) to investigate the changes in motion-induced magnetization recovery when introducing Ge into the Li_6_PS_5_I structure. NMR is able to directly sense the diffusive motions of the ^7^Li nuclei. By carrying out both laboratory-frame and rotating-frame SLR NMR experiments, we were able to uncover a range of Li^+^ motional processes that are usually hidden in data from alternating current (ac) impedance spectroscopy that is routinely used to characterize solid electrolytes. Moreover, by extending our measurements to the spin-1/2 ^31^P nucleus, we also use a constituent of the PS_4_^3–^ polyanions to study the magnetic dipolar spin fluctuations in crystalline Li_6+*x*_P_1–*x*_Ge_*x*_S_5_I. We show that ^31^P NMR is not only sensitive to diffusive phenomena of its own species but also informational with regard to Li ion translation through dipolar magnetic Li–P coupling effects. In combination with structural data from neutron diffraction and magic angle spinning (MAS) NMR, we show how site disorder opens a multitude of diffusion paths and how relaxation rates from NMR help in identifying the elemental jump processes contributing to overall ionic transport. Accordingly, our results illustrate how the differences in microscopic diffusional processes influence the macroscopic ionic conductivity; such insights, which relate local structures with results from atomic-scale diffusion measurements, are important to refine and scrutinize theoretical studies on this class of materials.

## Experimental Section

### Sample Preparation

The samples studied were prepared via a high-temperature solid-state reaction. Germanium sulfide (GeS, Alpha Aesar, 99.99% purity), phosphorus pentasulfide (P_4_S_10_, Merck, 99%), lithium iodide (LiI, ultradry Alfa-Aesar, 99.99%), and lithium sulfide (Li_2_S, Alfa-Aesar, 99.9%) were mixed in the appropriate stoichiometric ratio of the targeted composition and 3.5 wt % of lithium sulfide was added to improve purity of the final compounds. The mixtures were hand-ground in an agate mortar for 15 min, pelletized, and then added to quartz ampules, which were sealed under vacuum. The quartz tubes were carbon-coated and preheated at 800 °C under dynamic vacuum to remove all traces of water in the reaction atmosphere. The reactions were carried out at 550 °C for 2 weeks. After annealing, the obtained products were ground and used for the diffraction experiments and the various ^7^Li, ^6^Li, and ^31^P NMR measurements.

### Neutron Powder Diffraction

Neutron powder diffraction data were collected at Oak Ridge spallation neutron source (SNS, Oak Ridge National Laboratory) using the JANIS cryo-furnace at POWGEN diffractometer (BM11-A beamline).^41^ Approximately 3 g of each sample was loaded into an 8 mm diameter cylindrical vanadium container under inert atmosphere and sealed with a copper gasket to avoid air exposure during measurements. Using a single bank with a center wavelength of 1.5 Å, data were collected in high-resolution mode at 150 K (above the phase transition of Li_6_PS_5_I) and 200 K, respectively, for approximately 2 h. This bank allowed us to probe a *d*-spacing range from 0.5 to 6 Å with a resolution δ*d*/*d* < 9 × 10^–3^.^41^

### Rietveld Analysis

Rietveld refinements were carried out using the TOPAS-Academic V6 software package.^42^ The structural data obtained from neutron refinements of Li_6+*x*_P_1–*x*_Ge_*x*_S_5_I from Kraft et al.^17^ were used as starting model. The peak profile shape was described by a convolution of pseudo-Voigt and GSAS back–back exponential function. Fit indicators *R*_wp_ and goodness-of-fit (GoF) were used to assess the quality of the refined structural model. The following parameters were initially refined: (1) scale factor, (2) background (10 coefficients Chebyshev function), (3) peak shape, and (4) lattice parameters. After a good fit of the profile was achieved, the structural parameters were allowed to refine. Initially, (5) fractional atomic coordinates, (6) atomic occupancies, and (7) isotropic atomic displacement parameters were refined. Finally, lithium occupancy on other possible interstitial sites proposed by recent reports was investigated.^18,43,44^ The occupancy on these sites was constrained to the occupancy of the lithium positions proposed by Kraft et al.^17^ in order to maintain stoichiometry and electroneutrality. Output lithium thermal displacements were used as indicators for probing the authenticity of the eventual occupancy, where unphysical or negative values suggest the absence of lithium in the probed sites. The stability of the refinements was ensured by allowing to refine multiple correlated parameters simultaneously over several cycles. Finally, the crystallographic structures reported as CCDC 2142624, 2142625, and 2142626 were obtained by allowing the software to refine all possible structural parameters at the same time, further proving the stability of the simulated structure.

### Bond Valence Calculations

We carried out bond valence site energy estimations combined with a bond valence pathway analyzer using the softBV software tool developed by Adams and co-workers.^45,46^ The software calculates the surface energies and gives information about the positions of interstitial sites and saddle points, as well as the topology and dimensionality of ion-migration paths and the respective migration barriers. We used the structural information obtained from our Rietveld refinements as input for the calculations. Li^+^ was chosen as the mobile ion, and the grid resolution was set to 0.1 Å. We visualized the data with the VESTA software package.^47^

### MAS NMR

^6^Li and ^31^P NMR spectra under MAS conditions were recorded with a 500 MHz Avance spectrometer (Bruker). We measured at Larmor frequencies of 73.6 and 202.4 MHz and referenced the spectra to CH_3_COOLi and CaHPO_4_ for ^6^Li and ^31^P, respectively. For the experiments, the rotation speed was set to 25 kHz for the 2.5 mm rotors and to 60 kHz for the 1.3 mm rotors. In all experiments, the bearing gas temperature was ca. 303 K. The spectra were recorded with a single-pulse sequence with pulse lengths ranging from 1.40–1.5 μs (180 W) for ^31^P and 1.40 μs (100 W) for ^6^Li. For the experiments at 60 kHz, the pulse length was divided by 2. Up to 64 scans were accumulated with a recycle delay of 5–20 s. Frictional heating or centrifugal forces seem to have no large impact on the ^31^P NMR spectra as revealed by consecutive measurements carried out for different spinning periods. Hence, we conclude that spinning under these conditions does not induce any further structural changes.

### Static NMR Measurements

All samples were flame-sealed in Duran glass tubes under vacuum to permanently protect them from any reaction with air or moisture. Static, that is, nonrotating, NMR measurements in the form of line shape and SLR experiments were recorded to derive information about jump rates and activation energies of the motional processes present in Li_6+*x*_P_1–*x*_Ge_*x*_S_5_I. Variable-temperature SLR NMR measurements were recorded in both the laboratory (1/*T*_1_) and the rotating (1/*T*_1ρ_) frames of reference using a Bruker Avance III spectrometer that is connected to a shimmed cryomagnet with a nominal magnetic field of 7 T. All measurements were carried out in the temperature range of 173–493 K with a ceramic high-temperature probe (Bruker). Experiments to record the SLR NMR rate 1/*T*_1_ in the laboratory frame were conducted via the well-known saturation pulse sequence.^48^ For the π/2 pulses, a power level of 200 W was used. The pulse lengths ranged from 2.0–2.24 μs depending on temperature *T*. The area under the free induction decays was plotted against the waiting time *t*_d_ to construct the magnetization transients *M*_*z*_(*t*_d_). These transients were fitted with stretched exponentials including the stretching parameter γ that describes the deviation from single exponential behavior. The resulting rates 1/*T*_1_ were plotted as log_10_(1/*T*_1_) versus the inverse temperature 1/*T*.

From this Arrhenius diagram we deduced both the activation energies *E*_a_ and the motional correlation rates 1/τ_c_ from the various SLR NMR rate peaks. 1/τ_c_ is within a factor of less than 2 identical with the mean jump rate 1/τ of the nucleus under investigation.^49^

The corresponding rates in the rotating frame of reference were measured with the spin-lock pulse sequence.^50−52^ A locking frequency ω_1_/2π of 20 kHz was applied for both the ^7^Li and the ^31^P NMR experiments. The pulse power ranged from 6.55–13.5 W for ^7^Li. Similar to the laboratory frame, the spin-lock 1/*T*_1ρ_ NMR rates were extracted from magnetization transients *M*_ρ_(*t*_lock_) with the help of suitable stretched exponential functions.^52^ For temperatures below 313 K and samples with a Ge content equal or larger than 25 at. %, the quality of the fits increased when we used biexponential functions to analyze the transient signals; see below.

## Results and Discussion

### Structure as Seen by Neutron Diffraction and NMR

#### Neutron Diffraction

**Figure 1:**
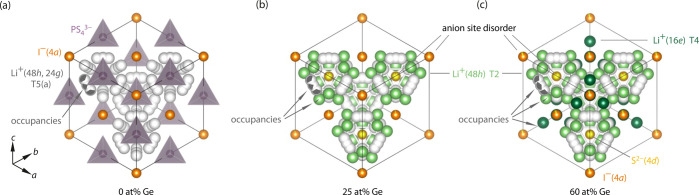
Crystal structure of Li_6+*x*_P_1–*x*_Ge_*x*_S_5_I for Ge content of (a) 0 at. %, (b) 25 at. %, and (c) 60 at. %. In the unsubstituted structure, the Li ions solely occupy T5(a) positions forming cagelike structures around the sulfur ions residing on Wyckoff 4*d* positions. When introducing Ge into the structure, slight anion site-disorder is observed for I^–^/S^2–^ on the positions 4*a* and 4*d*. Neutron diffraction reveals the occupation of new Li sites for the Ge-substituted samples. For 25 at. % Ge, the T2 positions (light green) close to the Li-rich cages are filled with low occupancies, as indicated in gray. For higher Ge content of 60 at. %, a second position T4 (dark green) in between the cagelike structures is revealed. The occupancy of these new sites forms a more interconnected Li substructure compared to the unsubstituted sample. In (b) and (c), the (P/Ge)S_4_-polyhedrons are omitted for better visibility of the Li substructure.

To set a basis for the investigation of the ion dynamics in the material class of Li_6+*x*_P_1–*x*_Ge_*x*_S_5_I, we thoroughly investigated the crystal structure by neutron powder diffraction and MAS NMR. To date, Li ions were only reported on type 5 (T5 and T5a) positions for Li_6_PS_5_I. As shown in Figure 1a, these Li-positions form cagelike structures around the nominal free sulfur positions (Wyckoff 4*d*). Partial occupation of a new Li-site T2 (Wyckoff 48*h*, see Figure 1b) was recently reported for Li_6_PS_5_Cl, Li_6_PS_5_Br,^43,44^ and Li_6+*x*_Sb_1–*x*_Si_*x*_S_5_I.^18^ Another position was found in Li_4.1_Al_0.1_Si_0.9_S_4_ (HT-Li_6.15_M_1.5_S_6_)^53^ and the respective oxygen-substituted sulfides,^54^ referred to as T4 (Wyckoff 16*e*); see Figure 1c. However, while Li^+^ occupation of nontype 5 sites has been experimentally observed in these “Li excess” argyrodites with stoichiometries *n*(Li) > 6, previous neutron diffraction studies of Li_6+*x*_P_1–*x*_Ge_*x*_S_5_I have assigned lithium ions to only occupy the type 5 sites.^43^

Therefore, inspired by these recent reports on other argyrodites, in this work the lithium substructure of the Ge-substituted argyrodites was reexamined. We employed Rietveld refinement against low-temperature, high-resolution neutron diffraction data for three selected compositions along the substitution series, that is, *x* = 0.0, 0.25, and 0.6, to study the subtle changes on the Li^+^ distribution brought up by the cationic substitution. According to the high ionic conductivity of these materials,^17^ low-temperature diffraction is of crucial importance to perform an accurate study of the lithium substructure. At lower temperatures, the lithium motion is severely reduced, therefore allowing the identification of new lithium positions that would be otherwise obscured at room temperature because of the highly delocalized lithium nuclear density of the neighboring sites. Here, neutron diffraction patterns were collected at 150 K for Li_6+*x*_P_1–*x*_Ge_*x*_S_5_I (*x* = 0.25 and 0.6) and at 200 K, that is, above the phase transition to the low-temperature phase, for Li_6_PS_5_I.^9,55^ The X-ray diffractograms collected at 300 K for all the compositions studied along the solid solution can be described well by the high-temperature cubic argyrodite polymorph crystallizing in the *F*43*m* space group. Refinements of the neutron diffraction data are reported in Figure S1. Together with the reflections belonging to the main argyrodite phase, additional minor contributions attributed to impurity phases (<5 wt %), such as LiI and Li_2_S, can also be observed.

The analysis of the neutron diffraction data shows that the substitution of Ge for P stabilizes the argyrodite cubic phase at lower temperatures. This evidence matches the results from other reports, where the substitution of P by Ge or Si in the halogen-free Li_7_PS_6_ argyrodite stabilizes the cubic phase at room temperature.^56^

**Figure 2:**
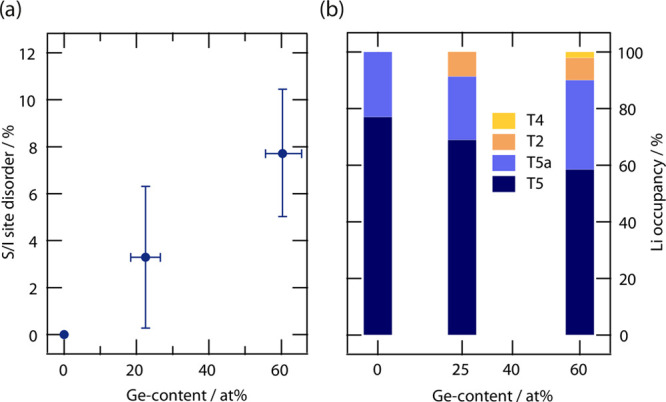
(a) Trend of the refined I^–^/S^2–^ anion site disorder for Ge fractions of 0, 25, and 60 at. %, in line with the literature values.^17^ (b) Lithium distribution for Li_6+*x*_P_1–*x*_Ge_*x*_S_5_I on the different sites as a function of Ge content. While for Li_6_PS_5_I the lithium occupancy was observed only for the typically reported type 5 positions, the Ge substituted samples show lithium occupancies for the newly explored type 2 and 4 sites, respectively.

Additionally, the Ge substitution expands the unit cell volume, due to the larger ionic radius of Ge^4+^ compared to that of P^5+^;^57^ a detailed structural information indicating the successful formation of stable solid solutions is shown in Figure S2. Figure 2a shows the increase of the I^–^/S^2–^ site disorder with the Ge content, which is in line with the literature values,^17^ therefore corroborating the accuracy of the refined structure and the good quality of the materials. The I^–^/S^2–^ site disorder is quite low (<8%) even for higher Ge content and especially compared to other argyrodite materials like Li_6_PS_5_X (X = Cl and Br).^58^

Neutron diffraction analysis also allows the precise analysis of the lithium substructure. Therefore, in this study the capacity of all possible interstitial sites offered by the anionic framework to host lithium was explored. Figure 2b shows the occupancy found for the various Li^+^ sites in Li_6+*x*_P_1–*x*_Ge_*x*_S_5_I as a function of Ge content. In contrast to Li_6_PS_5_I, that is, *x* = 0, where lithium occupies only type 5 and 5a sites, the solid solutions with *x* = 0.25 and 0.6 accommodate a fraction of lithium in type 2 tetrahedra. Moreover, for *x* = 0.6, additional occupancy was also found for type 4 sites (Figure 1c). It needs to be mentioned, however, that because of the low occupancy values (*g*(LiT4) < 3 ± 0.5%) the refinement of the lithium atomic coordinates of this position was unstable. Therefore, the atomic coordinates were not refined, and the lithium position was fixed approximately to the centroid of the type 4 tetrahedra. When fixing the position, the lithium occupancy, while being low, was still observable, indicating that the found lithium occupancy is real.

Lithium nuclear density was also observed on sites in close proximity of the type 4 tetrahedra for Li_6+*x*_Sb_1–*x*_Si_*x*_S_5_I, where Li^+^ occupies a position with all the atomic coordinates fixed by symmetry.^9^ These aspects indicate that the type 4 sites may be regarded as metastable position and are likely to take part in the ionic diffusion. Deiseroth and co-workers^55^ already pointed out the importance of the two interstitial Li-sites T2 and T4 on I-containing argyrodites. The two sites extend the localized Li^+^ substructure of Li_6_PS_5_I and are conveniently positioned in close proximity (T2) to the Li-cages and in between (T4); see Figure 1. For long-range ionic transport these positions need to participate in jumps with the T5 positions. Moreover, these results are supported by previous topological analyses via bond valence calculations that show the suitability of type 4 sites to host Li-ions and their importance for the ionic migration.^59^

A basic bond-valence calculation with the crystal structures obtained from our Rietveld refinements is shown in Figure S3. Comparing the unsubstituted Li_6_PS_5_I (*x* = 0) and the Ge-substituted Li_6.6_P_0.4_Ge_0.6_S_5_I, we can see that the newly occupied Li sites T2 and T4 are positioned directly on or in close proximity to spots of high Li density. Li ions residing on these positions help in constituting an interconnected diffusion network. They have access to jumps characterized by lower activation energies.

While the site energies of the T5(a) positions show only a little change, the newly occupied sites differ by 0.4–0.5 eV compared to the respective interstitial sites in the unsubstituted structures or in those with low Ge contents; see Table S1. While for T2 an increase in site energy is observed with regard to the originally interstitial site, position T4 turned out to be lower in energy. The same trend is also observed for the migration barriers. For the *intra*cage jumps the energy barriers are similar for all samples. However, for the jumps involved in *inter*cage diffusion, the barriers decreased with Ge-content; see Table S2. Additionally, the newly occupied sites T2 and T4 enable the ions to access new jump pathways. This fact clearly underpins the importance of these Li sites for overall ionic transport.

**Figure 3:**
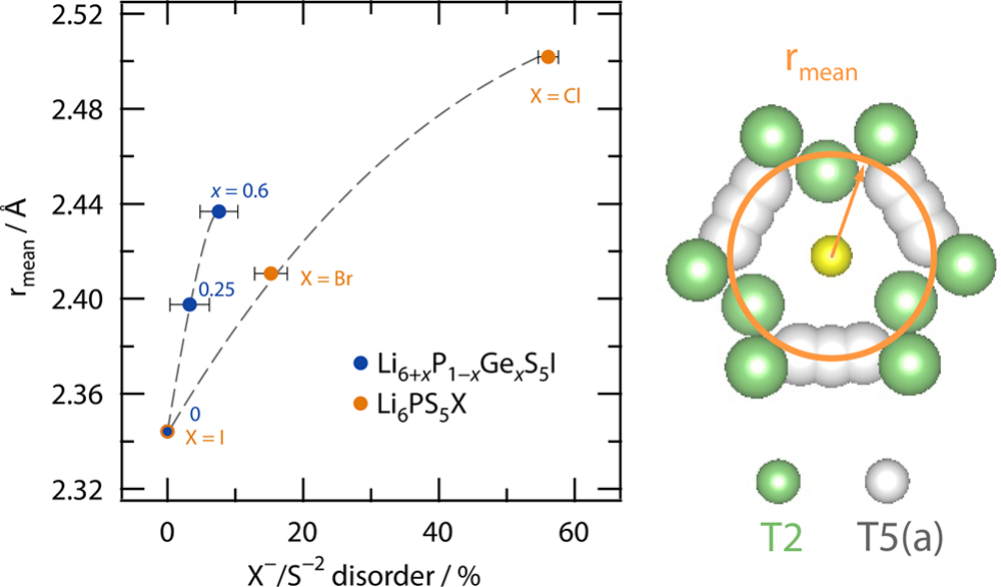
Average distance *r*_mean_ of the Li^+^ ions away from the center of the cage (nominal free sulfur position, Wyckoff 4*d*) as a function of the anion site–disorder for Li_6+*x*_P_1–*x*_Ge_*x*_S_5_I and Li_6_PS_5_X (X = Cl, Br, and I). With increasing disorder, the Li^+^ cages expand due to the smaller amount of anionic charge found at the cage center. Graphic representation of the mean distances of Li^+^ from the center of the cage, *r*_mean_.

To understand how the site–disorder and different lithium concentration affect the lithium distribution in a better way, Figure 3 shows the dependency of the average Li^+^ distance away from the center of the cage (i.e., nominal free sulfur position, Wyckoff 4*d*) against the degree of site disorder for Li_6+*x*_P_1–*x*_Ge_*x*_S_5_I and Li_6_PS_5_X. Due to the instability and the small lithium occupancy, the T4 position was not included in this structural analysis. The average Li^+^ distance away from the center of the cage, which can be seen as a measure of the size of the cage itself, correlates well with the site disorder in the structure. For both series the increasing site disorder results in an expansion of the lithium cage. This behavior can be explained by considering that a higher site disorder leads to a less average anionic charge density in the center of the cage as S^2–^ is replaced with X^–^. This change results in a weaker bonding environment for lithium and, therefore, in larger cages.

Comparing the trend shown in Figure 3 for the Li_6+*x*_P_1–*x*_Ge_*x*_S_5_I solid solution with the lower lithium content compositions, Li_6_PS_5_X, it is clear that the cages expand more for higher lithium concentrations. This behavior can be explained considering that the higher lithium content within the cage generates stronger Coulombic repulsion among the Li^+^ ions, allowing for both a faster expansion of the cage and *r*_mean_ if compared to the case of lower lithium concentration per formula unit. As theoretically suggested, the strong Li–Li Coulombic repulsion, generated by addition of extra lithium to the cage, results in an internal frustration that leads to rapid and correlated motion.^44^

#### ^6^Li and ^31^P MAS NMR

A complementary technique to study the chemical environments of specific ions more closely is given by MAS NMR. Here, we carried out experiments using the nuclei ^6^Li (spin-quantum number *I* = 1, 25 kHz spinning speed) and ^31^P (*I* = 1/2, spinning speeds of 25 kHz and 60 kHz), respectively. The ^6^Li NMR spectra (see Figure S4) show an asymmetric shape pointing to more than one contribution to the NMR signal. The ^6^Li nucleus (abundance 7.5%) is exposed to much lower (homo- and heteronuclear) magnetic dipolar and, most importantly, first- and second-order electric quadrupolar couplings than the ^7^Li one (abundance 92.5%). Ordinary MAS NMR is able to eliminate first-order interactions; second order-broadening effects remain untouched. Hence, ^6^Li NMR spectra benefit, in general, from improved spectral resolution. However, in the case of fast ion conductors rapid Li^+^ exchange processes at ambient temperature hinder the clear resolution of different crystallographic Li sites as coalescence effects dominate the spectra. Here, the line width decreases with higher Ge content, pointing to effective motional averaging processes of the residual couplings even under the MAS conditions applied. This trend is in full agreement with data from conductivity measurements.^17^

**Figure 4:**
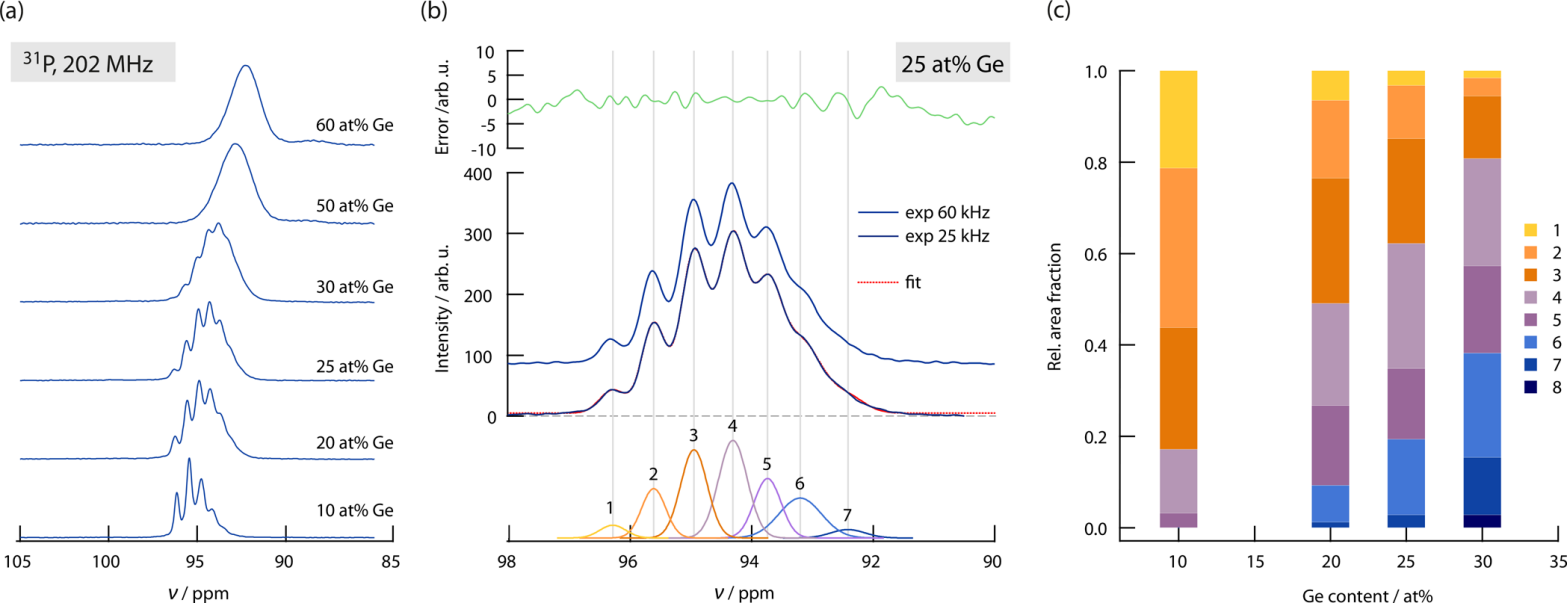
(a) ^31^P MAS NMR spectra (202 MHz) of Li_6+*x*_P_1–*x*_Ge_*x*_S_5_I with different Ge contents. Already at a Ge content as low as 10 at. %, the ^31^P spins sense distinct chemical environments. For a higher Ge content, the different distributions to the signal can no longer be resolved. (b) As an example, the deconvolution of the overall ^31^P NMR signal of Li_6.25_P_0.75_Ge_0.25_S_5_I is shown, which uses a combination of several Gaussian lines differing in positions on the ppm scale, amplitudes, and widths. Experimental data and fitting results are compared, together with the NMR measurement at 60 kHz. The upper part of the graph indicates the error between experimental and fitting values, the lower part shows the separate Gaussians shaped contributions to the overall ^31^P NMR signal. (c) Relative area fractions of the different Gaussian contributions to the overall ^31^P NMR signal for Ge contents of *x* = 0.1–0.3.

While the ^31^P NMR spectrum of Li_6_PS_5_I, recorded at a spinning speed of 25 kHz, is composed of only a single sharp line at 96.3 ppm (when referenced to 85% H_3_PO_4_),^7,8^ the sample with 10 at. % Ge does already show a multisplit NMR signal at the same rotation frequency. Thus, ^31^P MAS NMR clearly points to increased structural disorder within the Li_6+*x*_Ge_*x*_P_1–*x*_S_5_I as compared to Li_6_PS_5_I. Depending on the Ge substitution level, the overall ^31^P NMR signal consists of 5 to 8 separate lines; see Figure 4a. The higher the Ge content, the less visible the individual contributions to the total NMR line. Finally, a single broad line appears if the amount of Ge reaches levels larger than 30 at. %. Interestingly, at the same composition, Li_6+*x*_P_1–*x*_Ge_*x*_S_5_I reaches a limiting range for low activation energy as was derived from impedance spectroscopy data.^17^ To increase the spectral resolution, we applied MAS spinning speeds of up to 60 kHz. The coupling effects are, however, too strong to allow for an improvement in resolution, see Figure 4b.

To analyze the different NMR components to the overall signal we deconvoluted the spectra with a set of suitable Gaussian shaped functions; such a deconvolution is exemplarily shown in Figure 4b. Figure 4c shows the relative area fraction of the distinguishable ^31^P NMR signals for samples with a Ge content of up to 30 at. %. This analysis reveals that signals appearing at large ppm values decrease with larger Ge contents, while the spectral components located at lower ppm values increase. In samples with high Ge content, the ^31^P spin is subjected to a larger shielding effect and thus a lower local magnetic field resulting in an upfield shift. These shifts illustrate that coupling effects of ^31^P with less electronegative partners become more dominant. The same shift is also seen in ^6^Li NMR; see the Supporting Information.

Compared to previous ^31^P NMR investigations^14,60^ on Li argyrodites showing anion site disorder, the ^31^P lines measured here are very narrow covering a ppm range of only 3.8–5 ppm. This feature points to good crystalline compounds and/or a highly homogeneous external magnetic field *B*_0_ used to record the spectra. Wang et al.^14^ carried out a detailed ^31^P MAS NMR analysis to characterize the solid solution Li_6–*x*_PS_5–*x*_Br_1+*x*_. With increasing Br content, they observed the continuous occupation of the former S^2–^ site 4*d*. The ^31^P nucleus directly senses this change in chemical environment; the corresponding ^31^P NMR spectrum is composed of up to 5 different signals representing 0–5 bromine anions in its second coordination sphere. The width of the total ^31^P NMR signal in the study of Wang et al.^14^ is 8–8.21 ppm and, therefore, is larger than in the present case. The spectral distance between the lines turned out to be in the range of ca. 1 ppm (25 kHz), similar as observed in Figure 4. The very evenly spaced signals point to a magnetic coupling process of similar strength. It is also interesting to note that the spacing did not change when we increased the spinning speed from 25 to 60 kHz, which points to strong magnetic dipolar interactions that cannot be eliminated by such high rotation frequencies. Another interesting ^31^P MAS NMR analysis is found for Li_7_PS_6_ and Li_7_PSe_6_, for which the substitution of Se for S results again in five different chemical environments sensed by the ^31^P spins.^60^ The second coordination sphere is seen with shifts between the separate signals ranging from 1.58–2.35 ppm at 10–15 kHz spinning speed, while changes in the first coordination sphere (the P(S/Se)_4_ tetrahedra themselves) result in chemical shifts of >20 ppm.

In both studies random statistical anion distribution was observed for the second coordination sphere.^14,60^ In the present case, neutron diffraction tells us that for samples with 25 at. % or even 60 at. % Ge, anion site disorder is rather low taking values of only 3 and 7%, respectively (see Figure 2a). Thus, anion disorder cannot serve as an explanation of the multicomponent feature seen in ^31^P MAS NMR here. Additionally, electronegative S-rich environments, that is, deshielding effects, should dominate the ^31^P NMR chemical shift distribution. Thus, for such low extents of anion disorder, as is seen by neutron diffraction, we would expect a highly anisotropic distribution of NMR lines with the most intense signals being downfield-shifted, that is, appearing at large ppm values. Such features are, however, not observed here. Therefore, we suggest the different signals in the ^31^P spectrum arise from the substitution of P^5+^ by Ge^4+^, also explaining the shift of the signal toward lower ppm-values. In earlier studies the ^31^P nucleus is reported to be sensitive to the coordination sphere of position 4*d* at a distance of 4.4 Å. The next closest coordination sphere is at 5.1 Å with six nearest anion neighbors located at 4*a*. As mentioned above, the low degree of anion site disorder in the current structures cannot explain the broad distribution in the present ^31^P MAS NMR spectra. One argument to explain the occurrence of the various environments sensed by the ^31^P nuclei can be the electron density of the S^2–^ anion on the 4*d* position. This S^2–^ anion is tetrahedrally coordinated (bonding angle 109.47°) by 4 P or Ge atoms; see Figure S5. Considering that one bonding partner is fixed, because this ^31^P nucleus is detected in the MAS NMR experiment, the sulfur ion might hold 0–3 Ge partners on the remaining edges of the tetrahedral coordination sphere. With 4 of these sulfur ions surrounding the examined ^31^P nucleus, a large variety of electron densities on the sulfur position and, accordingly, on the central P nucleus is possible. While at lower Ge fractions the P–S bonds will statistically dominate, almost equal amounts of P and Ge in the structure will produce a large number of chemically quite similar environments leading to many overlapping signals, which explains the broad ^31^P NMR spectra of samples with large Ge contents (Figure 4a).

### Ion Dynamics as Probed by NMR

#### ^7^Li NMR Line Widths

Recording static ^7^Li NMR line shapes often yields the first insights in the Li^+^ self-diffusivity of ceramic ion conductors.^61^ Roughly speaking, NMR lines with widths in the order of several kHz are sensitive to motional processes with rates ranging from 10^4^ to 10^5^ s^–1^. At sufficiently low temperatures, Li^+^ ion exchange is slow, and the NMR line of a given ion conductor is determined by the various homo- and heteronuclear interactions leading to a broadened signal.^62^ With increasing temperature, however, these dipole–dipole couplings become increasingly averaged (motional narrowing) until at higher *T* a final NMR line is observed, whose width is determined by the nonaveraged interactions and the inhomogeneity of the external magnetic field *B*_0_.^62^

**Figure 5:**
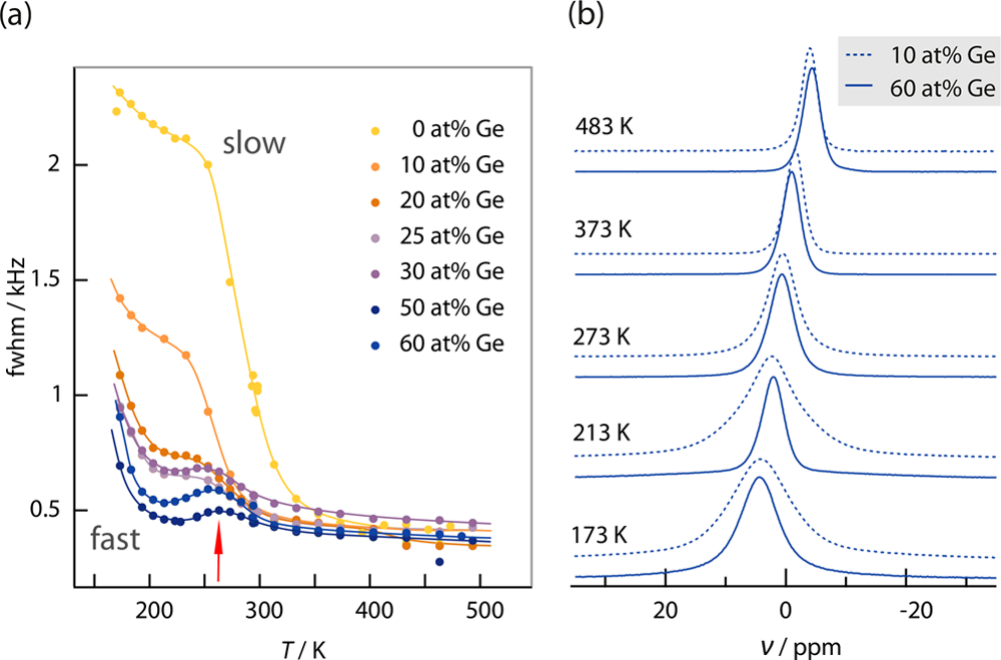
(a) Line widths (fwhm = full width at half-maximum) of static ^7^Li NMR lines (116 MHz) for Li_6+*x*_P_1–*x*_Ge_*x*_S_5_I with *x* ranging from 0–0.6. Data for the unsubstituted Li_6_PS_5_I (0 at. % Ge, *x* = 0) were taken from ref (7). The vertical arrow points to a local maximum that is caused by interactions of the mobile Li spins with paramagnetic centers. (b) Temperature dependence of the ^7^Li NMR line shapes of two samples (10 and 60 wt %) for selected temperatures. The NMR line shape is similar at temperatures above 273 K for both samples, while at lower temperatures, the higher Ge content leads to significantly narrower lines which is in agreement with a higher Li^+^ diffusivity.

Li_6_PS_5_X (X = I, Br, and Cl) was already the subject of earlier NMR line shape studies.^7^ The temperature dependence of the ^7^Li NMR line widths shows a peculiarity for Li_6_PS_5_I as compared to the Cl and Br analogues.^7^ While motional narrowing (MN) of the NMR lines of the latter two is governed by a single step, Li_6_PS_5_I shows narrowing in two steps and reaches the extreme narrowing regime at 100 K higher than its sibling compounds.^7^ This shift toward larger temperatures directly reveals the poor Li^+^ ion conductivity in the ordered Li_6_PS_5_I compound. In Figure 5a, only the second MN step is shown; the full curve, which also takes into account the phase transition of Li_6_PS_5_I, is published elsewhere.^7^

Here, such a two-step MN behavior is also observed for the Ge-substituted argyrodites Li_6+*x*_P_1–*x*_Ge_*x*_S_5_I, particularly for the samples with 10 and 20 at. % Ge, see Figure 5a. Again, only the second MN step is shown here, which is seen in a temperature range of 233–273 K. In the extreme narrowing limit, the line width reaches a value of ca. 400 Hz. Hanghofer et al.^7^ assigned the second narrowing to a slow Li^+^ motional process that only kicks in at higher temperatures. As long-range ionic transport in Li_6_PS_5_I is poor compared to Li_6_PS_5_Br and Li_6_PS_5_Cl, the second MN process was ascribed^7,17^ to the rate-limiting step referring to Li^+^ jump processes that connect the Li-rich cages in Li_6_PS_5_I.^63,64^ For Li_6_PS_5_Br and Li_6_PS_5_Cl, these processes occur with much higher probability shifting this MN step toward a much lower *T*. In the low-*T* regime, rapid *intra*cage Li^+^ hopping processes also contribute to the line-narrowing behavior. Even in Li_6_PS_5_I, these rapid but localized processes occur; however, because of their anisotropic nature, they cannot fully average the overall NMR line.

Considering the results shown in Figure 5a, we notice that the second MN process continually vanishes when going to samples characterized by higher Ge contents. Thus, the probability of *inter*cage Li^+^ jumps steadily increases with *x* enabling the Li^+^ ions to move over longer distances in Li_6+*x*_P_1–*x*_Ge_*x*_S_5_I. Ge substitution is thus responsible for the exchange processes that are able to effectively average Li–Li dipolar interactions.

Comparing the ^7^Li NMR lines of the samples with 10 at. % and 60 at. % Ge (see Figure 5b) shows that these differences in both line width and shape are most notably at low temperatures (≤273 K). While at high temperatures, that is, above 373 K, the lines are very similar, the Ge-rich sample is to be characterized by significantly narrower NMR lines at low temperatures. This fact agrees perfectly with the higher conductivity of Li^+^ in higher substituted samples as observed by impedance spectroscopy which probes the (average) macroscopic transport parameters.^17^

Before revealing further details of Li^+^ diffusivity by means of NMR relaxation measurements, we shall briefly discuss the local maximum (see arrow in Figure 5a) in motional narrowing seen for the samples with rather high Ge contents of 50 and 60 at. %, respectively. According to our knowledge, this behavior is typically seen for samples containing paramagnetic centers. As the ion hopping rate increases with temperature, the probability of the mobile ion encountering a paramagnetic center as nearest neighbor increases. Hyperfine interactions between the paramagnetic center and the Li spin will alter the precessional phase of the mobile Li^+^. If the maximum time between these encounters is shorter than the effective transverse (spin–spin) relaxation time *T*_2,eff_(*T*), then the NMR line will broaden again. With increasing temperature, the effective fluctuating rate will match the perturbation frequency resulting in a maximum. Further narrowing occurs when the residence time of the mobile ion close to the paramagnetic center becomes very short. Altogether, the MN curve passes through a local maximum that is due to the interaction of the diffusing Li spins with paramagnetic centers. The shape of the ^7^Li NMR line width may sensitively depend on the number fraction of paramagnetic centers present. Effects as those described above were also observed for concentrations as low as 0.03%.^65,66^

#### ^7^Li NMR Spin–Lattice Relaxation Experiments

**Figure 6:**
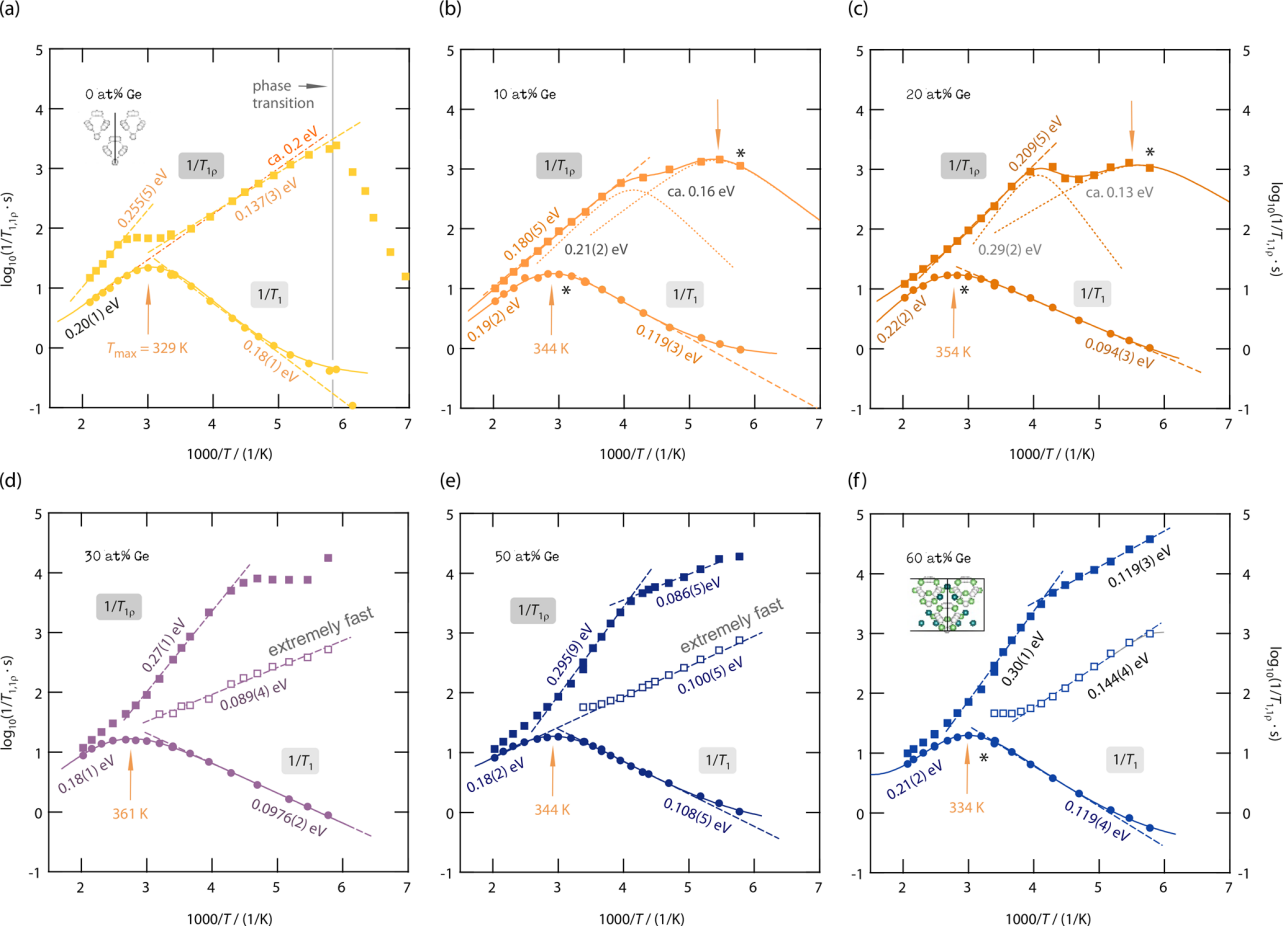
Arrhenius plots of the (diffusion-induced, *T* > 200 K) ^7^Li NMR SLR rates 1/*T*_1_ (circles, 116 MHz) and 1/*T*_1ρ_ (squares, 20 kHz locking frequency ν_lock_) for Li_6+*x*_P_1–*x*_Ge_*x*_S_5_I with Ge contents of (a) 0 at. %, (b) 10 at. %, (c) 20 at. %, (d) 30 at. %, (e) 50 at. %, and (f) 60 at. %. The data for the unsubstituted sample Li_6_PS_5_I (a) were taken from ref (7). Solid lines represent BPP (global) fits; dotted lines show separate contributions to the global fits. The dashed lines display linear fits of the low-*T* and/or high-*T* regions. For the rates 1/*T*_1_, the temperature at which the rates pass through a maximum is also indicated.

In contrast to NMR line shape measurements, ^7^Li NMR SLR measurements are sensitive to faster Li^+^ exchange processes with motional correlation times in the order of some nanoseconds.^49^ Provided that longitudinal NMR spin–lattice relaxation is solely induced by diffusional processes, the logarithm of the rate 1/*T*_1_ will pass through a maximum if plotted versus the inverse temperature, as is well-documented in the literature.^49,67^ In this temperature regime, any coupling of the spins with phonons or conduction electrons, (see Figure S7) is almost negligible.^68^ Here, for all samples a fully resolved ^7^Li 1/*T*_1_ NMR rate peak of asymmetric shape is detected (Figure 6a–f). The peak maximum occurs when the angular Larmor frequency (ω_0_ = 116 MHz × 2π for ^7^Li) is in the order of the mean correlation rate, ω_0_ ≈ 1/τ_c_.^49,69,70^ Hence, at *T*_max_, which is the temperature at which the rate passes through the peak maximum, the translational jump rate is in the order of 10^9^ s^–1^ which corresponds to specific conductivities in the mS cm^–1^ range.

Importantly, when studying the 1/*T*_1_(1/*T*) curves shown in Figure 6, distinct differences show up which need some discussion. First, *T*_max_ increases rather than decreases when part of the P^5+^ is exchanged by Ge^4+^ reaching a maximum value (361 K) for samples with 25–30 at. %. For even higher Ge contents, the maximum of 1/*T*_1_(1/*T*) shifts toward lower temperatures again, reaching almost the same value (328 K) as that for nonsubstituted Li_6_PS_5_I (*x* = 0). Second, the peaks of samples with Ge contents of 25–30 at. % appear to be broader than those seen for *x* = 0.

As we will discuss also below, the rate peak of Li_6_PS_5_I, characterized by no anion site disorder, is almost solely caused by fast, localized *intra*cage ion dynamics.^7^ It appears at quite a low temperature and is symmetric in shape. We assume that this peak is also present in samples with larger Li content but superimposed by new peaks appearing at slightly higher or even lower temperatures leading to a broader overall 1/*T*_1_(1/*T*) NMR response. This feature is best seen for the samples with 25–30 at. % Ge. As an example, the peak for the sample with 30 at. % Ge appears at *T*_max_ = 361 K and is, most likely, affected by both *intra*cage and *inter*cage exchange rates with values on the MHz time scale. Such high jump rates will lead to ionic conductivities in the order of some mS cm^–1^; indeed the ionic conductivity of this sample reaches values in the order of 0.1 mS cm^–1^.^17^ Hence, if at first glance the total response is understood as a superposition of several contributions, including also peaks appearing below the benchmark of 329 K of Li_6_PS_5_I, then the shift of the overall 1/*T*_1_(1/*T*) ^7^Li NMR peaks shown in Figure 6 is not in contradiction with an increasing ionic conductivity^17^ when going from *x* = 0 to *x* = 0.3.

The changes in *T*_max_ and peak width is also related to the change in amplitude *R*_max_ (≡ 1/*T*_1_(*T*_max_)) of the rate peaks. *R*_max_ decreases for low Ge substitution and increases again for higher values. While for increasing Ge substitution the Li position 24*g* gets more occupied, the occupancy of the 48*h* position, which is closer to the iodine ion, decreases;^17^ see also Figure 6b. This means that the Li–I heteronuclear interactions decrease, but at the same time, the homonuclear coupling of Li^+^ ions increases due to both the higher Li content and the occupation of new Li positions. For the intermediate substitution levels (20–30 at. %) the reduction in heteronuclear coupling seems to dominate and to cause *R*_max_ to decrease. In contrast, for higher Ge contents the occupancy of the new lithium positions T2 (new 48*h*) and T4 (16*e*) as well as the increased occupancy of 24*g* point to stronger dipolar and electric quadrupolar coupling constants, which explains the rise in *R*_max_. In the case of predominant quadrupolar relaxation, *R*_max_ is generally proportional to the square root of the nuclear quadrupole coupling constant.^71^

##### On the Asymmetry of the NMR Rate Peaks

A more quantitative analysis of the rate peaks is possible when evaluating their position and shape with appropriate spectral density functions to extract activation energies *E*_a_, attempt frequencies 1/τ_0_, and jump rates 1/τ. The Li jump rate is assumed to be thermally activated according to 1/τ = 1/τ_0_ exp(*E*_a_/*k*_B_*T*) with *k*_B_ being Boltzmann’s constant. In general, the motional correlation rate 1/τ_c_ is proportional to a spectral density function *J*(ω_0_) ∝ 1/*T*_1_(ω_0_,*T*) that contains the temporal spin fluctuations sensed by the SLR NMR experiments. Here, we used the Lorentzian-shaped function introduced by Bloembergen, Purcell, and Pound (BPP)^72−74^ to analyze the peaks 1/*T*_1_(1/*T*): *J*(ω_0_) ∝ τ/(1 + (τω_0_)^β^). β is the so-called asymmetry parameter that quantifies the deviation (β < 2) of a given peak from symmetric behavior (β = 2).^49,70,75^ Symmetric behavior is expected for uncorrelated motion or for materials for which short- and long-range diffusion are described by the same activation energy.^76^ While in the case of uncorrelated motion the underlying motional correlation function (*G*(*t*′)) is often a single exponential function, *G* deviates from simple exponential behavior when we have to deal with correlated dynamics.^49^ Correlated ion dynamics and/or structural disorder are frequently discussed as being the origins of a peak asymmetry.^49,77^ The parameters describing the BPP curves used to parametrize the 1/*T*_1_(1/*T*) peaks of Figure 6 are listed in Table 1. We modified the fits such that they also take into account the deviations of 1/*T*_1_ seen at very low temperatures (<200 K). These deviations, which were here approximated with a power law 1/*T*_1_ ∝ *T*^κ^ (1 < κ < 1.6),^51^ are caused by coupling of the Li spins with lattice vibrations and/or paramagnetic centers.^76,77^ In general, coupling of the spins with conduction electrons might play a role too in this low-*T* regime. Table 1 also includes *T*_max_, *R*_max_, and the amplitude *C* (∝ *R*_max_). Values of *C* in the range of 10^9^–10^10^ s^–2^ are typical for quadrupole interactions of ^7^Li. Furthermore, we analyzed the low-*T* flank of the 1/*T*_1_(1/*T*) peaks with a linear function that yields *E*_a,low_.

**Table 1:** Results of the BPP-Type NMR Relaxation Fits for the ^7^Li NMR 1/*T*_1_(1/*T*) Rate Peaks for the Samples with Varying Ge Contents (0–60 at. % Ge)^a^

compound	*E*_a_ (eV)	σ_330K_ (S cm^–1^)^c^	*E*_a,low_ (eV)	*C* (s^–2^)	β	1/τ_0_ (s)	*T*_max_ (K)	*R*_max_ (s^–1^)
Li_6_PS_5_I^b^	0.20(1)	8.8 × 10^–6^	0.18(1)	11(1) × 10^9^	1.92(1)	0.8(1) × 10^–12^	329	20.8
Li_6.1_P_0.9_Ge_0.1_S_5_I	0.19(2)	4.4 × 10^–5^	0.119(3)	8.6(4) × 10^9^	1.73(8)	1.5(8) × 10^–12^	344	17.8
Li_6.2_P_0.8_Ge_0.2_S_5_I	0.22(2)	1.3 × 10^–4^	0.094(3)	7.9(6) × 10^9^	1.46(5)	1.2(9) × 10^–12^	354	17.2
Li_6.25_P_0.75_Ge_0.25_S_5_I	0.21(2)	4.8 × 10^–4^	0.094(3)	8.0(2) × 10^9^	1.45(6)	2(2) × 10^–12^	361	17.3
Li_6.3_P_0.7_Ge_0.3_S_5_I	0.18(1)	1.6 × 10^–3^	0.098(1)	8.0(1) × 10^9^	1.56(5)	4(2) × 10^–12^	361	16.7
Li_6.5_P_0.5_Ge_0.5_S_5_I	0.18(2)	3.9 × 10^–3^	0.108(5)	8.7(4) × 10^9^	1.76(2)	3(2) × 10^–12^	344	18.8
Li_6.6_P_0.4_Ge_0.6_S_5_I	0.21(2)	1.2 × 10^–2^	0.119(4)	9.4(3) × 10^9^	1.8(1)	0.8(5) × 10^–12^	334	19.8

a The table includes the activation energies *E*_a_, coupling pre-factor *C*, asymmetry parameter β, and the inverse of the attempt frequency τ_0_ as well as temperature *T*_max_ and the amplitude *R*_max_ of the relaxation rate peak at the maximum. Moreover, we list the activation energies characterizing the low-*T* flank of the peaks. For comparison, ionic conductivity values σ recorded at 330 K are also shown. Electronic conductivities, as estimated via chronoamperometric polarization measurements, are by many orders of magnitude lower (10^–9^ S cm^–1^) and do not depend much on Ge content; see Figure S7. At room temperature, the ionic conductivity σ of Li_6.6_P_0.4_Ge_0.6_S_5_I turned out to be 5.4 mS cm^–1^ for a sample pellet in a cold-pressed state; sintering of the pellet results in an increase to 18.4 mS cm^–1^, as has been shown elsewhere.^17^

b NMR data taken from ref (7).

c Data taken from ref (17).

It turned out that *E*_a_ from the BPP fits take values of approximately 0.2 eV and deviate only little from sample to sample. The rates 1/τ_0_ show values in the order of 10^–12^ s as is commonly expected for phonon frequencies.^76,78^ As mentioned above and discussed earlier,^7,63^ in the case of Li_6_PS_5_I the symmetric 1/*T*_1_(1/*T*) peak (β = 2) is mainly governed by fast *intra*cage ion dynamics^79^ that do not lead to long-range ion transport. Its activation energy of 0.2 eV is in fair agreement with values deduced from MD simulations (0.26 eV)^63^ or bond valence approaches (0.15 eV).^64^ However, when going to samples including Ge and, thus, a larger number of Li ions, we notice that β decreases to values of only 1.45. The low-*T* flank of the peaks, which is sensitive to local (short-range) ion dynamics (ω_0_τ_c_ ≪ 1), reveals activation energies *E*_a,low_ only in the order of 0.1 eV characterizing the elementary jump processes.

This change in diffusion-induced 1/*T*_1_^7^Li NMR is already seen for the sample with only 10 at. % Ge and agrees with the behavior seen for the fast ion conductor Li_6_PS_5_Br.^7^ Since *E*_a,low_ remains quite constant for all different grades of substitution, only a low number of additional Li^+^ ions is sufficient to affect *E*_a,low_. Likely, correlated forward–backward jumps possibly involving Li^+^ exchange between the sites in the Li-rich cages and those connecting or near the cages, as the newly occupied 48*h* (T2) site, will contribute to the 1/*T*_1_ rate in this low-*T* regime of the samples. In other words, such new motional events, seen for *x* > 0, lead to an increase of the distribution width of the jump rate and to an increasing peak asymmetry. Hence, the exponent β, which is lowest for samples with ca. 25 or 30 at. % Ge (see Table 1), might serve as a parameter mirroring such distribution effects.

As the overall peaks become also broader in shape and flattened around *R*_max_ (Figure 6d), they can indeed be regarded as a superposition of relaxation processes caused by both *inter*- and *intra*cage ion dynamics. Due to the superposition and the resulting broad rate peak, the exclusive maximum for *intra*cage jumps, as seen for Li_6_PS_5_I, cannot be detected any longer. Instead, *T*_max_ represents now an average of different maxima. As the concomitantly seen processes cannot be separately resolved in the 1/*T*_1_(1/*T*) rate, the applied fit yields average dynamic parameters. We conclude that *inter*cage Li^+^ exchange is triggered or initialized by both (i) additional Li^+^ ions residing near or between the cages and (ii) the S^2–^/I^–^ anion site disorder. Besides anion disorder, as it has been discussed elsewhere,^17^ the occupation of originally empty Li^+^ sites might be the most important origin of the increase in overall Li^+^ translational dynamics observed by NMR.

Our interpretation of β in terms of distribution widths is corroborated by the fact that for structurally ordered Li_6_PS_5_I (*x* = 0), a completely symmetric peak is seen (β = 2). In poorly conducting Li_6_PS_5_I, the *inter*cage jump processes are much less frequent and, thus, almost not captured by the low-*T* flank of the SLR NMR experiment. For the samples with very high Ge contents (*x* = 0.5 and 0.6), the distribution width of 1/τ_c_ slightly narrows again, reflected by an increase of β. At the same time, the peak shifts back to a position (*T*_max_ = 334 K for *x* = 0.6) that is similar to that of Li_6_PS_5_I (*T*_max_ = 329 K, *x* = 0). Hence, the peak seems to be increasingly governed by the fast *intra*cage jump processes. The 1/*T*_1ρ_ NMR experiments will reveal that the high ionic conductivity of the sample with *x* ≥ 0.3 can be explained by the rather fast spin-lock NMR relaxation, which is more sensitive to long-range ion transport, see below.

In contrast to *E*_a,low_, on the high-temperature side (*E*_a,high_) of a given peak (ω_0_τ_c_ ≪ 1) the SLR NMR experiment does also probe less frequent Li^+^ jumps; that is, the jumps between sites connected by a higher activation barrier. In general this flank is sensitive to Li^+^ diffusion processes on a longer length scale; in the easiest cases, it directly reflects the activation energy that is probed by conductivity measurements in the low frequency limit (*E*_a_ = *E*_a,cond._).^25,49^ The activation energy derived from the BPP analysis is identical to that in the limit ω_0_τ_c_ ≪ 1, *E*_a_ = *E*_a,high_.^49,75^ In the present case, extremely rapid motional events might also contribute to the high-*T* flank leading to lower activation energies than those probed by macroscopic measurements, which are, in the low-frequency limit, sensitive to through-going ion transport. As mentioned above, *E*_a_ does not vary much with *x*, taking values from 0.18 to 0.21 eV; see Table 1.

For comparison, we evaluated some elementary diffusion pathways the Li^+^ ions have access to through the new positions in the Ge-containing samples. Our bond valence estimations yield energy barriers for T5(a)–T2 of 0.197 eV (25 at. % Ge) and 0.188 eV (60 at. % Ge), only slightly depending on Ge-content. These values support those extracted from the BPP-type NMR fits; see Table 1. According to the bond valence calculation, the *intra*cage jumps between the very close T5(a) sites are to be characterized by very low activation energies ranging from 0.049–0.062 eV for all samples; see Table S2. Also, these ultrafast jumps are likely included in the diffusion-induced rate peaks seen in 1/*T*_1_ NMR. The variations in *E*_a_ do also support our idea of dealing with superimposed NMR peaks, as discussed above.

As mentioned before, the occupation of new Li sites allows for additional translational Li^+^ jump processes. Jumps between the sites T5(a) and T4 in the sample with 60 at. % Ge are characterized by a value of 0.253 eV. These jump processes, being crucial for the connection of the separate Li-rich cages, are expected to be characterized by lower rates. Accordingly, a different NMR technique is required to detect them.

##### On the Detection of Slower Ionic Motion

The dynamic sensitivity window of ^7^Li NMR SLR rate measurements can be expanded from the MHz to the kHz regime by using the spin-lock SLR technique that measures transversal relaxation of the magnetization in the presence of a weak *B*_1_ field.^52,80^ Such 1/*T*_1ρ_ experiments are, per se, sensitive to motional processes occurring on a longer length scale. Hence, the method is indeed useful to probe long-range ion transport. The diffusion-induced 1/*T*_1ρ_ NMR rates of all samples are included in Figure 6. In general, for a given 1/*T*_1_ NMR peak probed at a Larmor frequency in the MHz range, a corresponding 1/*T*_1ρ_(1/*T*) peak is expected to appear at much lower temperature *T*_max_.^49,81^ In general, while for 1/*T*_1_ the rate peak shows up if the condition ω_0_ ≈ 1/τ_c_ is fulfilled, for the spin-lock peak, the condition ω_1_ ≈ 1/τ_c_ is valid, with ω_1_ ≪ ω_0_ resulting in *T*_max,ρ_ ≪ *T*_max_.^49^

Starting with Li_6_PS_5_I, when coming from low temperatures the ^7^Li 1/*T*_1ρ_(1/*T*) NMR rates first increase. Below the phase transition temperature of 160 K, Li^+^ ion diffusivity is extremely poor in Li_6_PS_5_I, and this increase reflects the low-*T* flank of a 1/*T*_1ρ_(1/*T*) peak of this modification of Li_6_PS_5_I.^7^ Above 160 K, the rates enter the regime of a high-*T* flank. Most likely, this flank corresponds to the 1/*T*_1_(1/*T*) peak seen at 329 K. Accordingly, it seems to be also influenced by the *intra*cage ion dynamics. Interestingly, at higher temperatures, 1/*T*_1ρ_ increases again and passes through a local maximum at ca. 360 K. As has been shown recently, this shallow peak reflects the *inter*cage ion dynamics.^7,17^ Introducing 10 at. % Ge and increasing Li from *x* = 0 to *x* = 0.1 has a drastic effect on the spin-lock 1/*T*_1ρ_ response. Peaks at temperatures much lower than 250 K appear. We assume, as shown for site-disordered Li_6_PS_5_X (X = Cl and Br),^7^ that various *inter*cage (and *intra*cage) Li^+^ diffusion processes induce 1/*T*_1ρ_ SLR in this *T* range. In Figure 6b,c, the 1/*T*_1_ and 1/*T*_1ρ_ rate peaks corresponding to each other are marked by asterisks. For the samples characterized by 10 and 20 at. % Ge, respectively, the 1/*T*_1ρ_ rate peak observed at low temperatures shows a slight temperature shift that reflects the one already seen for 1/*T*_1_.

**Figure 7:**
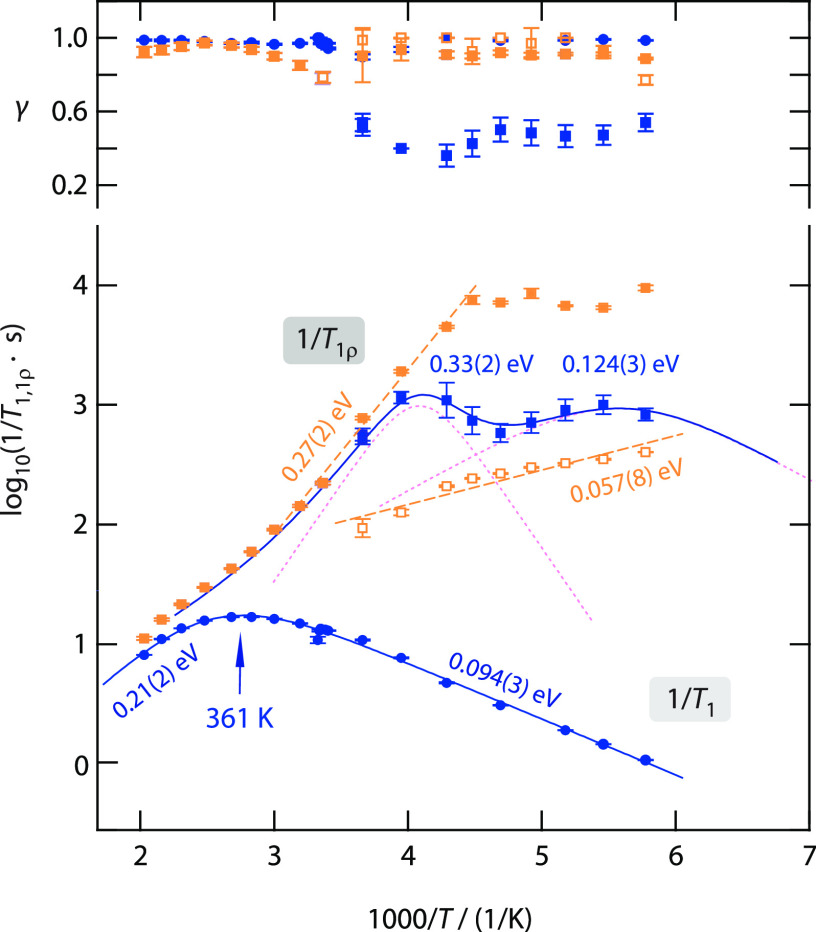
Arrhenius plot of the diffusion-induced ^7^Li NMR SLR rates 1/*T*_1_ (circles) and 1/*T*_1ρ_ (squares) of Li_6.25_P_0.75_Ge_0.25_S_5_I (25 at. % Ge). Solid lines represent BPP global fits; dotted lines show the separate contributions to the overall response. Dashed lines mark linear fits of the low-*T* or high-*T* flanks. The upper part of the graph shows the stretching coefficients γ of the stretched exponential fits used to parametrize the magnetization transients. The lines serve here as guides to the eye. For 1/*T*_1ρ_, the two different fits including error bars are shown: Orange squares refer to the rate when fitting the transients by a simple exponential decay function, and blue squares indicate the two rates from using a biexponential expression.

Importantly, when considering samples with even higher Ge contents, the 1/*T*_1ρ_ NMR rates display an interesting new feature. The spin-lock magnetization transients *M*_ρ_(*t*_lock_) of the samples with Ge contents of ≥25 at. % need to be parametrized with a sum of two exponential functions resulting in two relaxation times for each experiment at temperatures below 330 K (1000/*T* ∼ 3). Figure 7 compares the relaxation rates of the sample containing 25 at. % Ge when analyzing the transients with a stretched single or a biexponential function. As indicated by the respective error bars, fitting via biexponentials functions yields the higher accuracy in this case. Accordingly, the transients reveal a second relaxation process, which is clearly resolved if the Ge content reaches values of 30 at. % and higher; see Figure 6d–f. A similar behavior has already been reported for the fast ion conductor Li_6_PS_5_Br, which is structurally characterized by both Li^+^ and anion disorder.^7,10,43^

The distinct relaxation processes seen in spin-lock NMR are thermally activated by different activation energies. We notice that with increasing Ge content the high-*T* flank of one of the two 1/*T*_1ρ_ NMR rates shows relatively high activation energies. Such high energy barriers likely mirror long-range ion transport in the materials; thus, Li jump processes connecting the Li-rich rages in Li_6+*x*_P_1–*x*_Ge_*x*_S_5_I. Activation energies of 0.3 eV agree well with those measured by impedance spectroscopy;^17^ see Figure 6e,f and Table 2. Most likely, this magnetization component is to a non-negligible extent also influenced by spin–spin relaxation rather than by relaxation in the rotating-frame of reference. In the case of Li_6.3_P_0.7_Ge_0.3_S_5_I (see Figure 6d), the rates level off at *T* = 225 K taking a value of 8 ms which is expected for the spin–spin relaxation time *T*_2_ in this temperature regime. The further increase of this rate upon cooling would be in agreement with the increase in line width at very low temperatures, as presented in Figure 5a. However, the absence of the high-*T* MN step, which is seen for samples with large amounts of Ge, would correlate with the change of this spin-lock NMR component; see Figure 6e,f.

**Table 2:** Activation Energies *E*_a_, Prefactors, and β Values Derived from the BPP-Type Fits Used to Parametrize the Spin-Lock ^31^P NMR 1/*T*_1ρ_(1/*T*) Peaks Seen at Higher *T*^a^

Ge content (at. %)	*E*_a_ (eV)	*E*_a,imp_ (eV)	1/τ_0_ (s^–1^)	β	*E*_a__,low_ (eV)
0	0.44(4)	0.38	4(3) × 10^–12^	1.65	0.29(1)
10	0.29(2)	0.38	7(3) × 10^–11^	1.80	0.16(2)
30	0.17(1)	0.30	4(1) × 10^–9^	2, fixed	
60	0.13(1)	0.24	7(4) × 10^–10^	ca. 1.2	

a The activation energies *E*_a,low_ refer to the low-*T* slope only. Activation energies from impedance measurements, *E*_a,imp_, are also included, see ref (17).

The 1/*T*_1ρ_ NMR relaxation process seen down to very low temperatures probes extremely fast spin fluctuations that are characterized by activation energies ranging from 0.1 to 0.15 eV. The associated component of this *M*_ρ_(*t*_lock_) component is displayed in Figure 6e,f by using unfilled symbols. For Li_6.5_P_0.5_Ge_0.5_S_5_I, this flank can also be regarded as the high-*T* flanks of the corresponding 1/*T*_1_ NMR rate peaks; see the corresponding dashed line. The same interpretation has been used to explain the full NMR response of Li_6_PS_5_I,^39^ whose 1/*T*_1ρ_ NMR rates, if not analyzed separately from the 1/*T*_1_(1/*T*) peak (0.137 eV), can be understood as the flank of the corresponding 1/*T*_1_(1/*T*) peak (0.2 eV, see the dashed dotted line in Figure 6a).

In contrast, for 30 and 60 at. % the slopes of the flanks at low temperatures (see open squares in Figure 6e,f) are too low to correspond to the rate peaks seen in 1/*T*_1_ NMR. This is different from the behavior of Li_6_PS_5_Br and Li_6_PS_5_Cl where so-called joint fits considering both 1/*T*_1ρ_ and 1/*T*_1_ yield reasonable results.^7^ We recognize that the dynamic situation probed here for highly conducting Li_6+*x*_P_1–*x*_Ge_*x*_S_5_I is different to that seen for the Ge-free counterpart Li_6_PS_5_Br. We assume that the thermally weakly activated rates 1/*T*_1ρ_ (open symbols in Figure 6e,f) seem to be largely influenced also by the rapid *intra*cage ion dynamics. Hence, the biexponential behavior of 1/*T*_1ρ_, that is, most likely, also influenced by *T*_2_ effects, can be understood in terms of a bimodal Li^+^ diffusion behavior of *inter*cage (ca. 0.3 eV) and *intra*cage (0.1–0.15 eV) ion dynamics in argyrodite-type Li_6+*x*_P_1–*x*_Ge_*x*_S_5_I. Activation energies as low as 0.1 eV seen in 1/*T*_1ρ_^7^Li NMR point to extremely fast spin fluctuations which agree with the high ion conductivity seen for the sample with *x* ≥ 0.5. Such rapid ion dynamics in Li_6.5_P_0.5_Ge_0.5_S_5_I manifests itself in the fact that the peak maximum of 1/*T*_1ρ_(1/*T*) is expected to appear at very low *T*. In agreement with results from impedance spectroscopy, the activation energies deduced from 1/*T*_1ρ_ NMR turned out to be slightly larger for the sample with *x* = 0.6 (0.119 and 0.144 eV) compared to that characterizing the situation for *x* = 0.5 (0.086 and 0.097 eV).

##### ^31^P Spin–Lattice Relaxation

Another approach to investigate the ion dynamics in solid electrolytes can be monitoring the SLR behavior of immobile species like the ^31^P nuclei.^11,39,40,82^ As ^31^P is part of the PS_4_^3–^ polyanion, it is able to sense (i) the tumbling or rotations of these units and (ii) the dipolar spin fluctuations in its direct vicinity that are caused by the mobile ^7(6)^Li spins. The latter can be used to indirectly sense Li ion dynamics, as ^31^P acts here as silent observer of the Li translational dynamics.^39^ Here, we investigated three different compositions, namely, *x* = 0.1, 0.3, and 0.6, for Li_6+*x*_P_1–*x*_Ge_*x*_S_5_I and compared the results with the ^31^P NMR rates of Li_6_PS_5_I. We do not observe any spin-dilution effect, one might expect when P is continuously replaced by Ge. Such effect could impact the hetero- and homonuclear interactions to which ^31^P is subjected.

**Figure 8:**
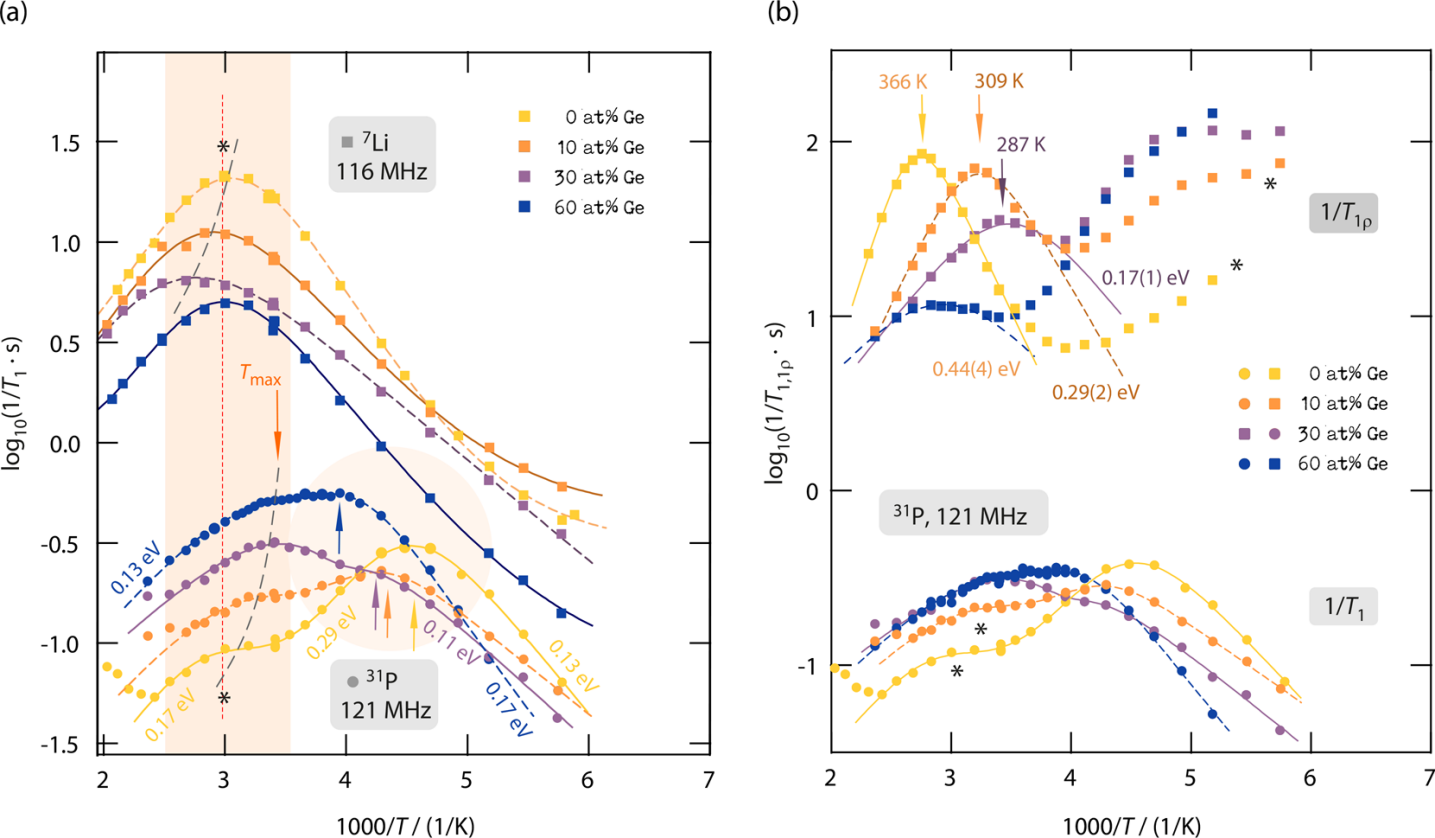
(a) Arrhenius plots of the diffusion-induced ^31^P and ^7^Li SLR NMR rates 1/*T*_1_ of Li_6+*x*_P_1–*x*_Ge_*x*_S_5_I with Ge contents *x* of 0, 10, 30, and 60 at. %, respectively. Circles show the rates for the ^31^P spin (121 MHz) and squares refer to the corresponding ^7^Li NMR ones (116 MHz). The curves were shifted by using a *y*-axis offset for the sake of better visibility. For 1/*T*_1_, the high temperature rate peaks in ^31^P SLR NMR mirror part of the overall SLR processes in ^7^Li NMR. In contrast, the rate peak at lower temperatures, which is best seen for *x* = 0 (0 at. % Ge), represents a relaxation process exclusively seen in ^31^P NMR. (b) Spin-lock ^31^P SLR NMR (1/*T*_1ρ_, 20 kHz) rates (squares) for the same Ge-contents. At temperatures above 250 K, we observed a distinct rate peak in 1/*T*_1ρ_ representing a very slow relaxation process to which the ^31^P nuclei are subjected. This process, especially for *x* = 0, is characterized by a steep slope on the low-*T* side, pointing to high activation energies that decrease for increasing Ge content. Also, for higher amounts of Ge, the peak shifts toward lower temperatures, revealing the influence of additional Li^+^ ions, the change in lattice parameters, and anion site disorder. For comparison, the ^31^P NMR 1/*T*_1_ rates are also shown without offset (circles), *vide supra*. Solid and dashed lines represent (global) BPP-type fits; see the text for further explanation.

Starting with the 1/*T*_1_^31^P NMR measurements, we observed two ^31^P NMR relaxation rate peaks instead of a single asymmetric one as seen for ^7^Li, *vide supra*, see Figure 8a. For *x* = 0, the ^31^P NMR rate peak located at higher temperatures corresponds in position with that seen in in ^7^Li NMR.^11^ For the Ge-substituted samples, the ^31^P NMR relaxation rate peaks are shifted toward slightly lower *T*. We parametrized these ^31^P NMR rate peaks with BPP-type fits to derive the activation energies listed in Table 3. The fact that the activation energies derived from ^31^P NMR are all smaller than the ones from the BPP-type fit of the ^7^Li 1/*T*_1_ NMR rates can be explained by two reasons. First, the ^31^P NMR response can be blurred by the overlap with the second rate peak at lower temperatures. Second, the ^7^Li NMR 1/*T*_1_ rate is likely a superposition of different jump processes and the ^31^P spins might be more sensitive to some of the diffusion processes rather than being able to sense all Li^+^ diffusion processes in equal shares. The latter explanation also explains the fact that *T*_max_ of these ^31^P NMR rate peaks do not exactly match with the position of the ^7^Li responses; see Figure 8a.

**Table 3:** Summary of Activation Energies *E*_a_ Derived from BPP-Type Fits of the 1/*T*_1ρ_ and 1/*T*_1_^31^P NMR SLR Rates^a^

Ge content (at. %)	*E*_a_ (*T*_1_, BPP) (eV)	*E*_a_′ (*T*_1_, BPP) (eV)	*E*_a,low_ (*T*_1_) (eV)	*E*_a_ (*T*_1ρ_, BPP) (eV)
0	0.17(2)	0.20(2)	0.13(1)	0.44(4)
10	0.13(2)	0.20(2)	0.09(1)	0.29(2)
30			0.11(1)	0.17(1)
60	0.13(1)	0.29(7)	0.17(1)	

a The values *E*_a_′ refer to the 1/*T*_1_^31^P NMR peak seen at lower *T*; see Figure 8a. *E*_a,low_ indicates the activation energies of the low-*T* flank.

The second rate peak of ^31^P 1/*T*_1_ NMR occurring at lower temperatures shows the relaxation of the phosphorus independent of Li^+^ diffusion because no corresponding phenomenon is seen in ^7^Li NMR relaxation. We assume that the PS_4_^3–^ units, as they do not share corners or edges with other structural elements, are able to perform small-step rotational jump processes.^11^ With increasing Ge content structural site disorder is, however, introduced. This site disorder slows down ^31^P NMR relaxation.^11,40^ At the same time, the shift of the ^31^P NMR peaks toward higher *T* brings the translational and rotational rate maxima closer together. As the Larmor frequencies of the nuclei (116 MHz for ^7^Li and 121 MHz for ^31^P) are close to each other, the two kinds of motion proceed on almost the same time scale for the site-disordered samples. In the case of Li_6_PS_5_I, the two modes are dynamically decoupled as the corresponding *T*_max_ markedly differ from each other. This behavior changes when going to samples with site disorder and very high ionic conductivities. For the sample with 60 at. % Ge, the strongest overlap of these two processes is probed (see Figure 8a), underlining the importance of translational–rotational couplings for overall ionic transport. A similar trend was observed for Li_6_PS_5_Br and Li_6_PS_5_Cl, recently.^11^ Answering the question whether the GeS_4_^4–^ units do also undergo rotational events is beyond the scope of the present study. As an example, Valakh et al. reported rotations in Cu_2_Zn(Sn_1–*x*_Ge_*x*_)S_4_ using DFT calculations.^83^ In another study by Nazar and co-workers rotations in Na_11_SnP/SbS_12_ were studied by AIMD simulations and MEM on neutron diffraction data.^37^ They found that PS_4_ rotations are facilitated, while SbS_4_ rotations are hindered in the respective structure. Calculations of the Helmholtz free energy revealed higher barriers for the S ligands of the SbS_4_ anion group compared to PS_4_. A similar trend might be observable for GeS_4_ polyhedra. As ^73^Ge is a spin-9/2 nucleus with a natural abundance of only 7.67%, spin–lattice relaxation NMR is not a suitable method to detect this kind of rotations. Further studies are required to elucidate this matter.

To finalize our analysis, we also carried out spin-lock 1/*T*_1ρ_^31^P NMR experiments to probe even slower, that is, rate-limiting steps of Li^+^ diffusion processes; see Figure 8b. While at high temperatures, the 1/*T*_1ρ_^31^P NMR rates pass through distinct rate peaks with rather slopes indicating high energy barriers sensed by the spins (see Figure 8b), at low temperatures, the rates increase again, reflecting the high-*T* flanks of the 1/*T*_1_ peaks discussed before. Furthermore, in the low-*T* regime the rates might be influenced by the ^7^Li spin fluctuations sensed by spin-lock ^7^Li NMR. Thus, a complex superposition of several processes governs the overall 1/*T*_1ρ_ SLR NMR response of ^31^P in this *T* range.

The ^31^P NMR relaxation peaks seen at high *T* are the most important ones useful for a comparison with data from impedance spectroscopy.^17^ We observe that the rate maxima shift toward lower temperatures with increasing Ge content. Simultaneously, the activation energies, taken from appropriate BPP fits, decrease significantly from 0.44 to 0.17 eV when increasing the Ge content from 0 to 30 at. % Ge; see Table 3. The low amplitude of the sample with 60 at. % Ge, likely a result of the low P-content of this sample, hinders extraction of a reasonable value for the activation energy. The other values found are, however, in quite good agreement with those measured by impedance spectroscopy that yielded values ranging from 0.38 to 0.24 eV;^17^ see Table 2. This agreement assists us in determining the origin of the spin-lock ^31^P NMR rate peaks seen at higher *T* in Figure 8a. Obviously, they represent the rate-limiting step of *inter*cage ion dynamics being essential for overall ionic transport.

Further evidence for this view is provided by comparing the ^7^Li NMR response with the ^31^P NMR relaxation rates, see Figure S6. The 1/*T*_1ρ_^31^P NMR rate peaks have direct corresponding peaks in 1/*T*_1ρ_^7^Li NMR, as is best seen for Ge-free Li_6_PS_5_I (Figure S6a). As the spin-lock experiments of ^7^Li and ^31^P were carried out with the same locking frequency of 20 kHz, the nuclei-dependent relaxation phenomena occur on the same time scale showing that the ^31^P relaxation NMR is indeed sensitive to rather slow Li^+^ motions.

Finally, the shape of the spin-lock ^31^P NMR peaks reveals that correlated motion is present in site-disordered Li_6+*x*_P_1–*x*_Ge_*x*_S_5_I. Values for the asymmetry parameter β range from 2 to 1.65. Moreover, relatively high prefactors and thus attempt frequencies are obtained in combination with large activation energies *E*_a_ (Table 2). This relation is known as the empirical Meyer–Neldel rule,^84,85^ which was also observed in semiconductor science.^86^ Notably, as mentioned above, ^31^P might not be sensitive to all spin-fluctuations the Li spins are subjected to. In particular, whereas we regard the ^7^Li response as a superposition of several jump processes that broaden the rate peaks, ^31^P acts as a selective observer for only some of the Li^+^ translational processes. Here, it seems to be highly sensitive to the rate-limiting step^39^ determining the long-range ion transport properties of the Li_6+*x*_P_1–*x*_Ge_*x*_S_5_I series, particularly including the sample with *x* = 0. This rate-limiting steps includes *inter*cage ion dynamics involving the newly occupied Li sites T2 and T4 between these cages.

## Discussion and Summary

To understand the enhanced ionic transport in Ge-substituted Li_6+*x*_P_1–*x*_Ge_*x*_S_5_I, we carefully reinvestigated the distribution of Li^+^ in the argyrodite framework by neutron diffraction at low temperatures. For 25 at. % Ge, we observed Li^+^ ions residing on the T2 lattice site (Wyckoff 48*h*), and for 60 at. % Ge, we also observed them on the T4 site (Wyckoff 16*e*) with small occupancies (see Figure 1). Additionally, to a relatively low degree also anion site disorder was observed for I^–^/S^2–^. These structural changes results in larger Li-rich cage structures than observed for the nonsubstituted sample (Figure 3). Calculations suggest that this expansion causes internal (geometric) frustration^87^ finally leading to rapid and correlated Li^+^ motion.^44^

Changes in the local structure were also detected by ^31^P MAS NMR; see Figure 4. A highly anisotropic chemical environment was observed already for the sample containing only 10 at. % of Ge. For Ge contents higher than 30 at. %, the anisotropy was too large to distinguish the separate contributions even if measurements were carried out at a spinning speed of 60 kHz. Our data shows that ^31^P MAS NMR is not only sensitive to anion site disorder^14,60^ but also able to detect substitution effects of cations such as Ge^4+^.

**Figure 9:**
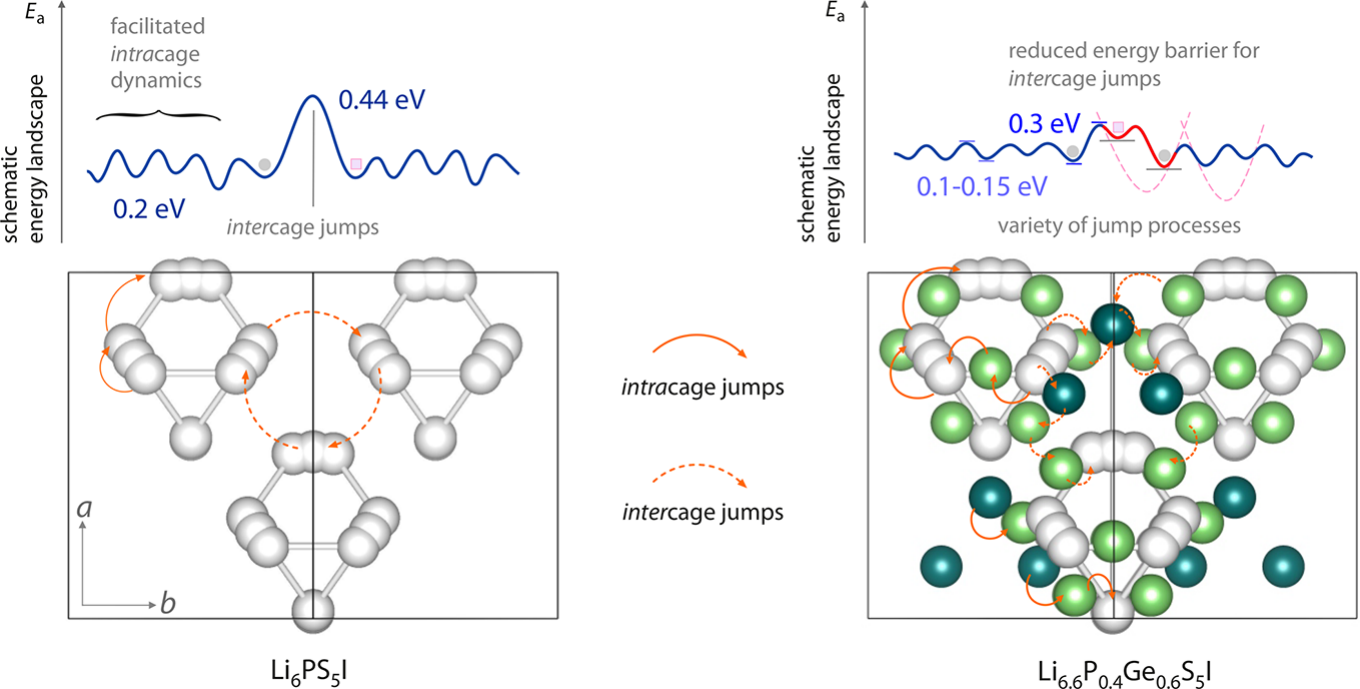
Distribution of Li^+^ ions in the crystal structure of Li_6+*x*_P_1–*x*_Ge_*x*_S_5_I with 0 at. % Ge (left) and 60 at. % Ge (right). Arrows indicate the available jump processes for the cations. A schematic energy landscape is also shown for each structure. In Li_6_PS_5_I, the Li^+^ ions in the cages are separated by a rather large barrier drastically slowing down the long-range ion transport. Filling the Li sites in between the cages reduces local energy barriers and widens the distribution of activation energies. Consequently, fast ion transport is realized in Li_6+*x*_P_1–*x*_Ge_*x*_S_5_I with sufficiently high Ge contents.

Variable-temperature ^7^Li and ^31^P NMR SLR measurements, carried out in both the laboratory and in the rotating frame of reference, were used to investigate the impact of structural site disorder on ion dynamics on different length scales, including the elementary jump processes at the atomic scale; an illustration of the main results is shown in Figure 9. 1/*T*_1_ rates point to very low activation energies in the order of ca. 0.1 eV (and even below) for all samples (see Figure 6). These rates are sensitive to very fast exchange processes with frequencies in the order of 10^9^ s^–1^. We ascribe these hopping processes to localized *intra*cage ion dynamics present in all samples of the series Li_6+*x*_P_1–*x*_Ge_*x*_S_5_I.

In the low-*T* limit, 1/*T*_1_ NMR is sensitive to only few jump processes within the time period set by the inverse Larmor frequency. On the high-*T* side of a given NMR rate peak, hundreds of jump events are probed during one spin precessional motion.^49^ Hence, long-range motion is probed that also senses larger barriers connecting the Li sites of an irregular potential landscape.^24^ If we deal with polycrystalline samples with micrometer-sized crystallites, as it is the case here, then the effect of blocking grain boundaries is, however, not seen in NMR spectroscopy. Such effects are usually probed by macroscopic techniques such as impedance or conductivity spectroscopy in the low-frequency limit.^88,89^ These methods can probe through-going ion transport.^90^

Li^+^ diffusivity on a longer length scale was detected by ^7^Li 1/*T*_1ρ_ rates measured at locking frequencies of 25 kHz. In spin-lock NMR, the Larmor frequency in the MHz range is formally replaced by a locking frequency in the kHz range. Hence, the time window, which is given by the inverse of this frequency, is enlarged by ca. 3 orders of magnitude.^49^ Again, the technique is still a microscopic one that is sensitive to bulk properties if we deal with micrometer-sized crystallites. Also, local jump processes are accessible with this technique.

For samples with high Ge-levels spin-lock ^7^Li NMR revealed several dynamic processes that start to govern the rates already at very low temperatures. These spin-fluctuations are, however, not seen for samples with a Ge content lower than 30 at. %. Below ambient conditions, we detected a second spin-reservoir that is subject to rather fast diffusion processes with low activation energies ranging from 0.089 to 0.144 eV (see Figure 6). Moreover, spin-lock NMR clearly revealed dynamic processes, whose activation energies of 0.3 eV agree with those usually seen in impedance spectroscopy being sensitive to long-range transport in the bulk. We ascribe these spin-fluctuations to mirror the *inter*cage ion exchange processes between the Li-rich cages. For Ge-bearing samples with *x* < 0.3, these processes need higher temperatures to become activated.

In the case of these samples with low Ge content, ^31^P NMR helped us to characterize the rate-limiting step for long-range ion transport in Li_6+*x*_P_1–*x*_Ge_*x*_S_5_I. In fact, ^31^P SLR NMR appeared to be more sensitive to the motion of Li^+^ than observing the mobile cation itself. We clearly see that upon Ge incorporation the corresponding spin-lock ^31^P NMR rate peaks shift toward lower temperatures; see Figure 8. At the same time, activation energies decrease from 0.44 eV to values lower than 0.2 eV indicating that the incorporation of Ge clearly influences the rate-limiting step for Li-ion conduction.

In summary, for Li_6+*x*_P_1–*x*_Ge_*x*_S_5_I, the large distribution of jump processes is initialized by the occupation of new Li sites and through the loss of Li^+^ site preference in samples with high Ge content. In Figure 9, we illustrate schematically how the schematic energy landscape changes when going from Li_6_PS_5_I to Li_6+*x*_P_1–*x*_Ge_*x*_S_5_I. In all compounds the Li^+^ ions have access to fast hopping processes within the Li-rich cages. These *intra*cage dynamics are characterized by high jump rates and low activation energies. In Li_6_PS_5_I, these regions of rapid local ion dynamics are, however, separated by rather large energy barriers significantly hindering long-range ion transport. Filling the Li-sites in between the cages reduces local energy barriers and widens the distribution of activation energies (Figure 9). Accordingly, rapid *inter*cage jump processes facilitate a boost in long-range, that is, through-going, ion transport. For *x* = 0.6, the partially occupied Li sublattices, remembering the idea of frustrated geometry, allow the Li^+^ ions to quickly diffuse through the sample without any energetically nonpreferential rate-limiting events.

In general, Figure 9 is the result of the important combination of reliable structural data, here yielding site occupancies of both the Li^+^ ions and the anions, with length-scale dependent information on cation dynamics, as both ^7^Li and ^31^P NMR can provide.

Much further work is, however, needed to fully understand ion dynamics in the argyrodites already known and in those awaiting discovery. In general, time-domain SLR NMR, with its relatively simple but informative experiments, will help in completing the pictures on Li^+^ ion dynamics if based on fragmentary data. Making length-scale-dependent information available is the crucial point for the development of safe and powerful all-solid-state lithium batteries that indubitably need ceramic electrolytes with outstanding Li^+^ diffusion properties.

## Conclusions

Li_6+*x*_P_1–*x*_Ge_*x*_S_5_I serves as a suitable model system to study the change in lattice properties, Li-content and site disorder on Li-ion transport and diffusivity. We used a complementary set of NMR techniques to probe both the elementary steps of ion hopping and the dynamic parameters characterizing long-range ion transport in the argyrodite-type framework.

By means of neutron powder diffraction combined with ^31^P MAS NMR, we studied structural changes upon substitution of Ge^4+^ for P^5+^. Most importantly, neutron diffraction revealed a continuous population of interstitial sites with Li^+^ ions with increasing Li content. At the same time, the extent of anion site disorder increases, however, to a much lesser extent. At 25 at. % Ge, Li^+^ ions start to occupy the new site T2 (Wyckoff 48*h*); at 60 at. % Ge, they also reside on the T4 position (Wyckoff 16*e*). The occupation of these originally empty sites forms an interconnected Li^+^ sublattice offering a larger variety of jump processes.

With the help of ^7^Li and ^31^P spin–lattice relaxation NMR, we were able to differentiate between the local *intra*cage dynamics and the *inter*cage jumps. In addition to commonly employed impedance spectroscopy providing an average value characterizing ionic transport, time-domain NMR methods, either carried out in the laboratory (*T*_1_) or rotating frame of reference (*T*_1ρ_), are able to differentiate between the various jump processes on the atomic scale. While very low activation energies in the order of ca. 0.1 eV (and even below) were ascribed to localized *intra*cage ion dynamics in Li_6+*x*_P_1–*x*_Ge_*x*_S_5_I, Li^+^ diffusivity on a longer length scale has to be described by activation energies of 0.3 eV, which is best seen for samples with *x* = 0.3, 0.5, and 0.6. In these samples, rapid spin fluctuations associated with *inter*cage ion exchange processes increasingly govern spin-lock ^7^Li NMR 1/*T*_1ρ_. At the other end of the compositional range of Li_6+*x*_P_1–*x*_Ge_*x*_S_5_I series, ^31^P NMR helped us to characterize the rate-limiting step in samples with *x* < 0.3.

The population of interstitial Li sites in Li_6+*x*_P_1–*x*_Ge_*x*_S_5_I, forming a variety of Li pathways connecting the Li-rich cages is responsible for the significant increase in Li ion conductivity in this class of argyrodite-type materials. Partly filling the Li sublattices goes along with a loss in Li^+^ site preference and a reduction of the relevant energy barriers governing macroscopic Li^+^ transport. Taken together, the present study provides a unified understanding of lithium motion in materials with argyrodite-type frameworks.

## Supporting Information Available

Rietveld refinements against neutron powder diffraction data, lattice parameters, bond valence calculations, ^6^Li MAS NMR spectra, Arrhenius plots of variable-temperature ^7^Li and ^31^P SLR NMR rates recorded simultaneously, polarization measurements to estimate electronic conductivities (PDF)

## Accession Codes

CCDC 2142624–2142626 contain the supplementary crystallographic data for this paper. These data can be obtained free of charge via www.ccdc.cam.ac.uk/data_request/cif, or by emailing data_request@ccdc.cam.ac.uk, or by contacting The Cambridge Crystallographic Data Centre, 12 Union Road, Cambridge CB2 1EZ, UK; fax: +44 1223 336033.

## Abbreviations

AIMD: *ab initio* molecular dynamics
NMR: nuclear magnetic resonance
MAS: magic angle spinning
MN: motional narrowing
SLR: spin–lattice relaxation
