# Supplementary material for: Opening Diffusion Pathways through Site Disorder: The Interplay of Local Structure and Ion Dynamics in the Solid Electrolyte Li6+xP1–xGexS5I as Probed by Neutron Diffraction and NMR
Source: J Am Chem Soc. 2022 Jan 20;144(4):1795–812. doi: 10.1021/jacs.1c11571 (PMC8815078; doi:10.1021/jacs.1c11571)
Supplement: Supplementary file 1 — ja1c11571_si_001.pdf [file ja1c11571_si_001.pdf]

Supporting Information

Opening Diffusion Pathways Through Site Disorder: The Interplay of Local Structure and Ion Dynamics in the Solid Electrolyte $Li_{6+x}P_{1-x}Ge_xS_5I$ as Probed by Neutron Diffraction and NMR

Katharina Hogrefe[a*], Nicolò Minafra[b], Isabel Hanghofer[a], Ananya Banik[b], Wolfgang G. Zeier[b,c], and H. Martin R. Wilkening[a*]

[a] Institute of Chemistry and Technology of Materials, Graz University of Technology (NAWI Graz), Stremayrgasse 9, A-8010 Graz, Austria.

[b] Institute of Inorganic and Analytical Chemistry, University of Münster, Correnstrasse 30, D-48149 Münster, Germany.

[c] Institut für Energie- und Klimaforschung (IEK), IEK-12: Helmholtz-Institut Münster, Forschungszentrum Jülich, Corrensstrasse 46, 48149 Münster, Germany.

* katharina.hogrefe@tugraz.at; * wilkening@tugraz.at

The Rietveld refinements for the neutron powder diffraction data for the argyrodites $Li_6P_{1-x}Ge_xS_5I$ (x = 0.0, 0.25, 0.6) are shown in Figure S1.

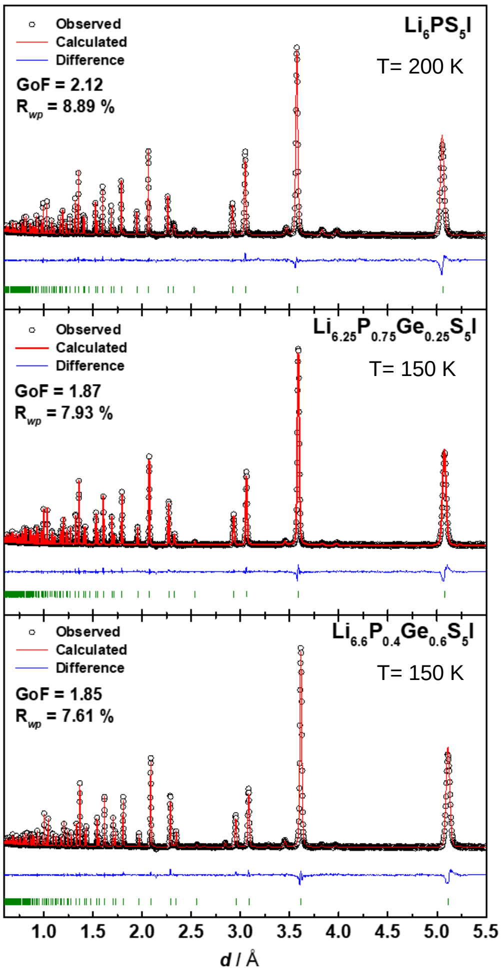

Figure S1. Exemplary Rietveld refinements against neutron powder diffraction data measured at 150 K and 200 K relative to $Li_{6+x}P_{1-x}Ge_xS_5I$ (x = 0.25, 0.6) and Li_6PS_5I, respectively. Experimental data are shown in black and the red line denotes the calculated pattern, whereas the difference profile is shown in blue. Positions of the argyrodite Bragg reflections are indicated by green vertical ticks. The low values for the fit indicators together with the flat profile difference confirm the good quality of the calculated structural model.

Besides stabilizing the cubic phase, the Ge substitution expands the unit cell volume, due to the larger ionic radius of Ge^{4+} compared to P^{5+}.[1] The lattice parameters obtained from Rietveld refinements of the three selected compositions for the low-temperature neutron studies (x = 0.0, 0.25, 0.6) are shown in Figure S2a, together with the lattice parameters obtained from profile fit of room-temperature X-ray data for all other studied compositions. Due to the lower temperature at which the diffraction experiments were performed, the lattice parameters carried out from neutron data are systematically smaller than the ones obtained from the room-temperature X-ray data. The linear increase of the lattice parameters upon substitution of Ge^{4+} by P^{5+} indicates the successful synthesis of stable solid solutions.

Further insights into the structural modifications brought up by the elemental substitution can be provided by Rietveld refinements. To corroborate the incorporation of the substituent, the occupancy of Ge on the nominal P site (Wyckoff 4*b*) was allowed to refine and the obtained values are reported against the nominal content in Figure S2b. The linear correlation between refined and nominal occupancy indicate the successful substitution that is further supported by the volume expansion of the $(P_{1-x}Ge_x)S_4$ tetrahedra upon Ge substitution (see Figure S2c).

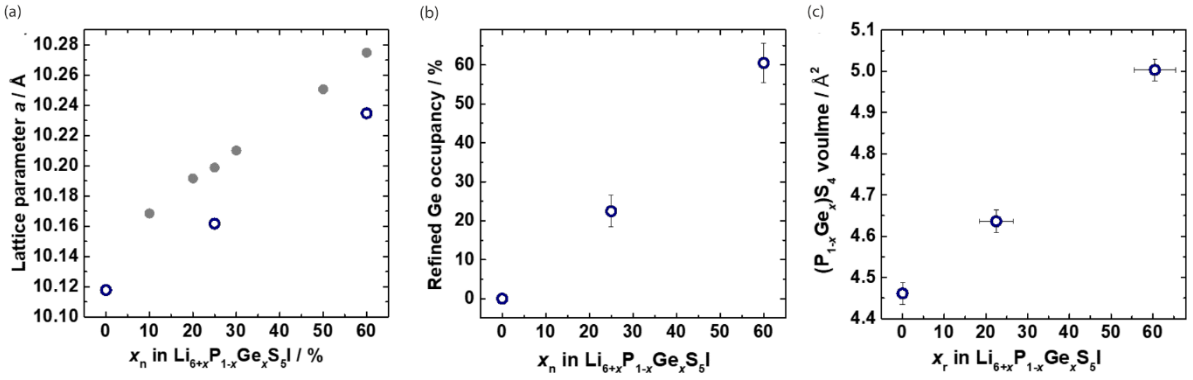

Figure S2. The linear increase of (a) the lattice parameters, carried out from (gray) X-ray and (blue) neutron data, together with (b) the refined Ge occupancy against the nominal Ge content x_n indicate the formation of a true solid solution. Moreover, (c) the expansion of the $(P/Ge)S_4$ units against the refined Ge content x_r corroborates the successful substitution of Ge for P in such tetrahedral environment.

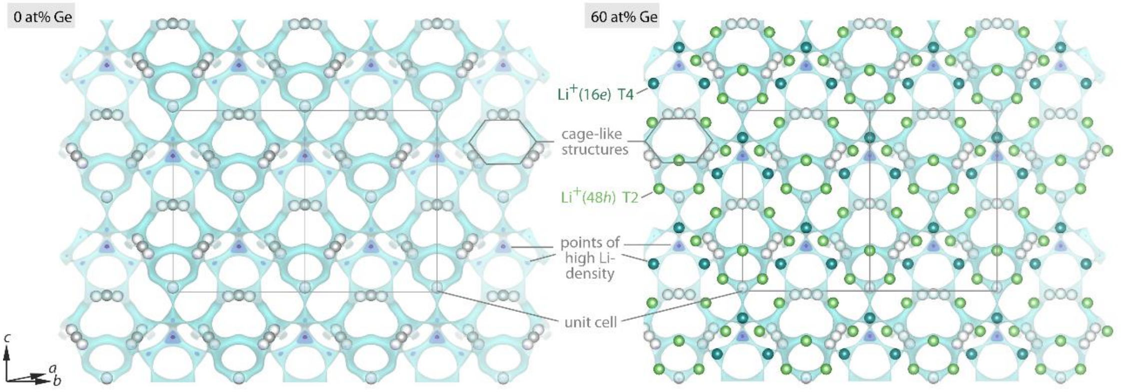

Figure S3. Bond valence calculations for the unsubstituted Li_6PS_5I (left) and the Ge-substituted $Li_{6.6}P_{0.4}Ge_{0.6}S_5I$ (right) showing only the Li-ions in the crystal structure. Note that newly occupied Li positions in the Ge-containing compounds are found on sites with high Li density on the migration path of Li also in Li_6PS_5I.

Table S1. Energy minimum of lattice sites for Li_6PS_5I and Ge-doped samples with 25 at% and 60 at% of Ge. Sites denoted with "T" are lithium occupied sites, interstitial sites are denoted with "i"

Site	Li_6PS_5I	$Li_{6.25}P_{0.75}Ge_{0.25}S_5I$	$Li_{6.6}P_{0.4}Ge_{0.6}S_5I$
T5(a)	0.174	0.195	0.190
i3/T2	0.310	0.378	0.363
i2/T4	0.216	0.207	0.177
i1	0	0	0

Table S2. Energy barriers for the different possible jump processes of the Li-ions as derived from the bond valence analyzer. The Li-sites are denoted with "T", interstitial points with "i" and saddle points with "s" The jump proceses are distinguished in jumps occuring within the Li-rich cages (*intra*cage) and those contributing to connecting them (*inter*cage). While the *intra*cage show little difference for the undoped and doped samples, the *inter*cage jumps show lower energy barriers and a larger varitey with increasing Ge-content.

	Sites	Li_6PS_5I	$Li_{6.25}P_{0.75}Ge_{0.25}S_5I$	$Li_{6.6}P_{0.4}Ge_{0.6}S_5I$
*intra*cage	T5(a)-s1	0.049	0.058	0.062
	T5(a)-s2/s3/s2-i3/T2	0.175	0.197	0.188
*inter*cage	T5(a)-s4-i2/T4	0.305	0.274	0.253
	i3/T2-s7/s5	0.586	0.578	0.497
	T4-s9-T2	-	-	1.059
	i1-s3-T4			0.397

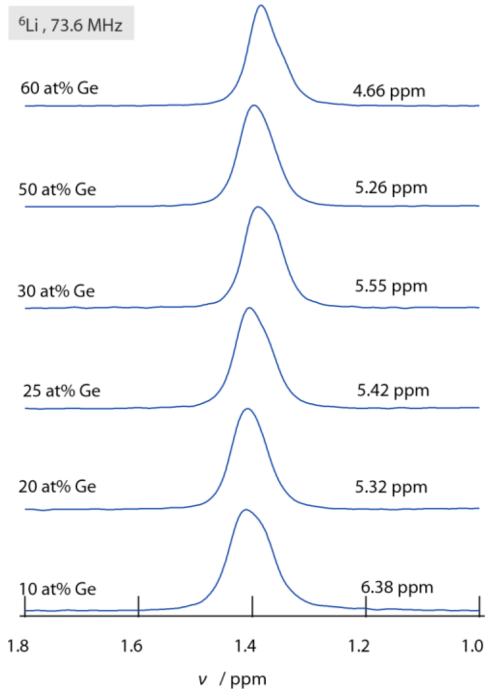

Figure S4. ^6Li MAS NMR spectra of $Li_{6+x}P_{1-x}Ge_xS_5I$ with different Ge-contents. Note the asymmetric shape of the ^6Li spectra pointing to several contributions to the signal.

With higher Ge-content the ^6Li lines measured under MAS conditions show a slight upfield shift, *i.e.*, to lower ppm-values. A similar trend is also observed for the ^{31}P MAS lines. It results from the fact that coupling of the respective nuclei with less electronegative partners becomes more dominant. As Ge^{4+} exchanges P^{5+} upon substitution and also additional Li^+ ions are incorporated, the chemical environment of the ^6Li species shifts upfield, resulting also in a small change in chemical shift in the respective spectra.

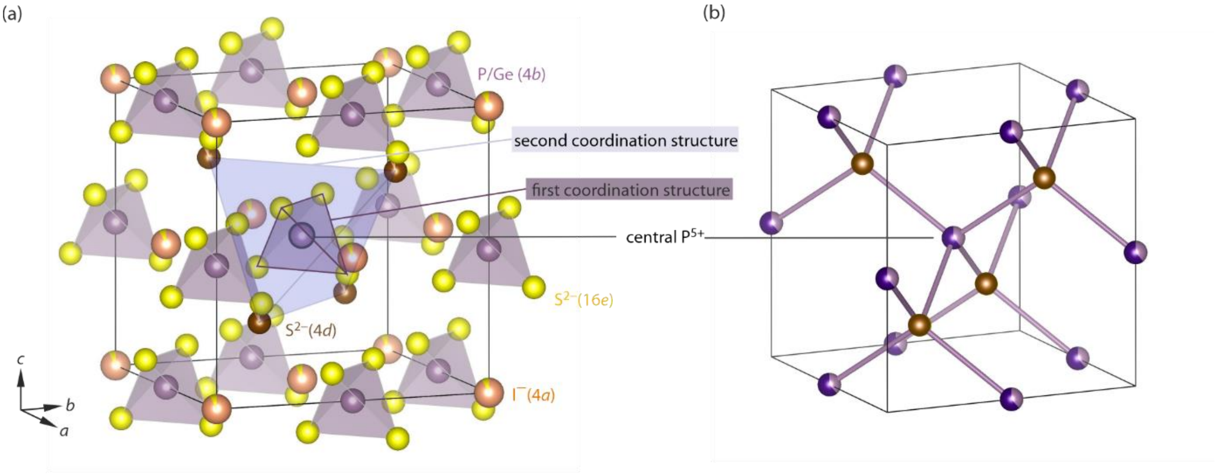

Figure S5. Cubic unit cell of space group $F\bar{4}3m$ for $Li_{6+x}P_{1-x}Ge_xS_5I$ with Li-ions omitted for better visibility of the P–S coordination. Left: sulphur in the 4d position (brown), S/I at position 4a (orange), PS_4 tetrahedra (purple and yellow). Right: Illustrating the coordination of the central P-ion coordinated by four sulphur ions (brown) on 4d positions. These sulphur ions are themselves coordinated by four P or Ge ions.

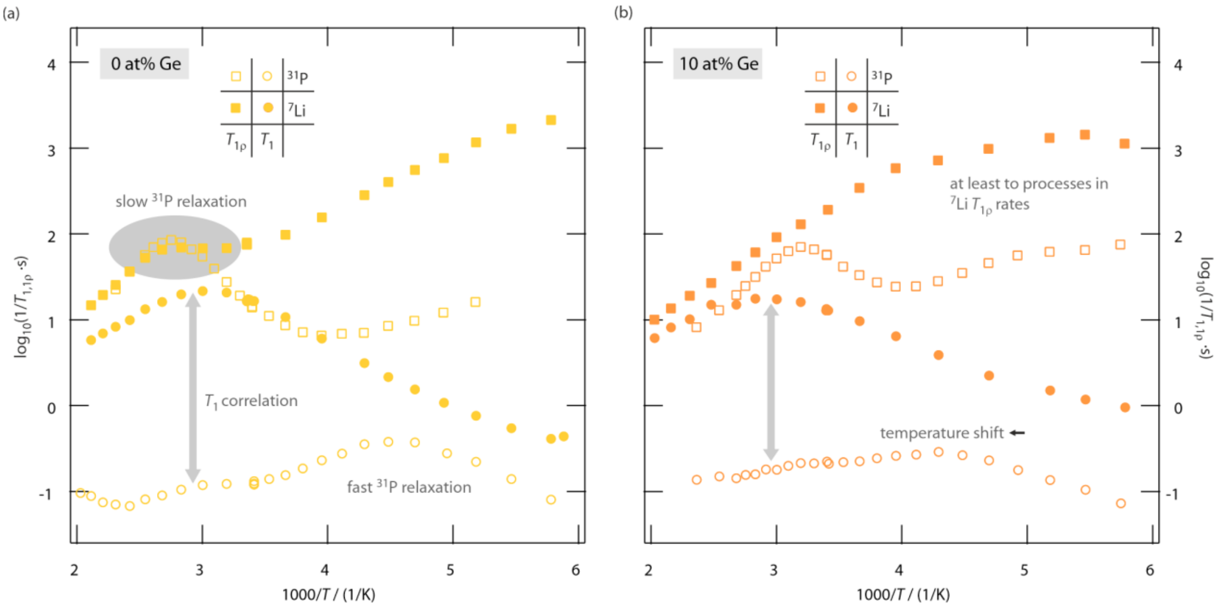

Figure S6. Comparison of the NMR spin-lattice relaxation $1/T_1$ (circles) and $1/T_{1\rho}$ (squares) of ^7Li (filled markers) and ^{31}P (empty markers) for the samples $Li_{6+x}P_{1-x}Ge_xS_5I$ with (a) 0 at% Ge and (b) 10 at% Ge, respectively.

Polarisation measurements. For the dc polarisation measurement, a pressed pellet (d = 8 mm; h=0.88mm (20 at%) 1.14 mm(60 at%) was sputtered with ion-blocking Au electrodes on both sides. The sample preparation was carried out under Ar atmosphere. We measured the pellet in an air-tight 2-electrode Swagelok-type cell connected to a Parstat MC potentiostat (Princeton Applied Research) equipped with a low-current option. The chronoamperometric polarization experiments were performed in a Faraday cage at ambient temperature for 7 days (604800 s, 10 s/point) with a potential set to 0.1 V.

For both samples the overall conductivity σ reaches a limit in the order of 10^{-9} Scm^{-1} which is due to the electronic contribution to the total conductivity. This value represents the upper limit of any electronic conductivity in the samples investigated. Here, σ_{eon} seems to be almost independent of the Ge-content in the investigated samples. However, considering the large difference in total conductivity between the samples with 10 at% and 60 at% Ge, the electronic contribution, that is, the respective transference number, is higher in the poor ionic conductor with 10 at% Ge.

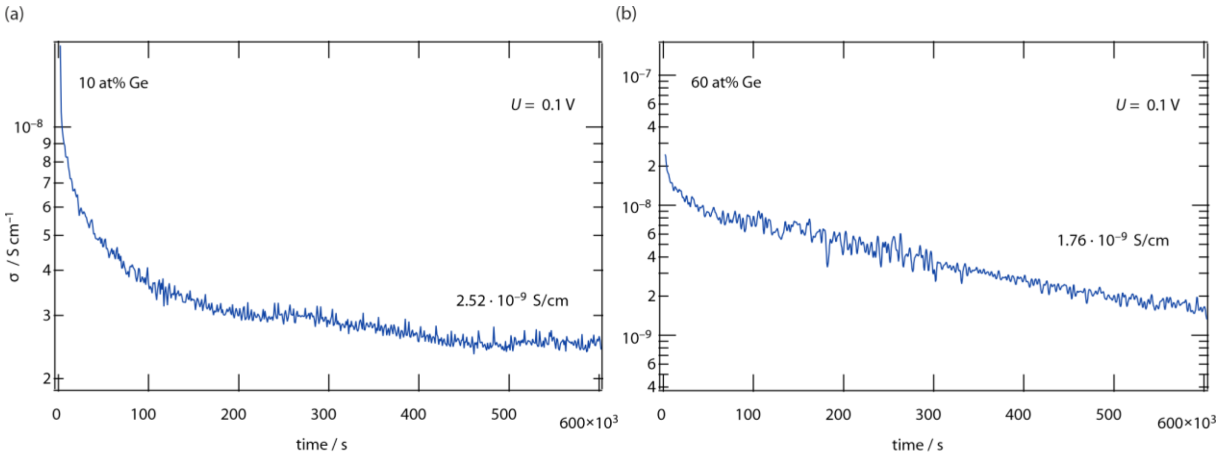

Figure S7. Chronoamperometric evolution of the overall conductivity over 7 days for for the samples $Li_{6+x}P_{1-x}Ge_xS_5I$ with (a) 10 at% Ge and (b) 60 at% Ge. The applied potential was set to 0.1 V. Both samples reach a limiting value at sufficiently long waiting time, which is identified to be governed only by electronic conductivity σ_{eon}. Here, σ_{eon} turned out to be in the order of 10^{-9} S cm^{-1} or lower.

References

1. Shannon, R. D., Revised effective ionic radii and systematic studies of interatomic distances in halides and chalcogenides. *Acta Crystallogr., Sect. A* **1976,** *32*, 751.
